# Galectin 3–binding protein suppresses amyloid-β production by modulating β-cleavage of amyloid precursor protein

**DOI:** 10.1074/jbc.RA119.008703

**Published:** 2020-01-29

**Authors:** Tsuneyoshi Seki, Motoi Kanagawa, Kazuhiro Kobayashi, Hisatomo Kowa, Naoki Yahata, Kei Maruyama, Nobuhisa Iwata, Haruhisa Inoue, Tatsushi Toda

**Affiliations:** ‡ Division of Neurology/Molecular Brain Science, Kobe University Graduate School of Medicine, Kobe, Hyogo 650-0017, Japan; § Department of Rehabilitation Science, Kobe University Graduate School of Health Sciences, Kobe 654-0142, Japan; ¶ Department of Anatomy I, Fujita Health University School of Medicine, Toyoake, Aichi 470-1192, Japan; ‖ Center for iPS Cell Research and Application (CiRA), Kyoto University, Kyoto 606-8507, Japan; ** Department of Pharmacology, Faculty of Medicine, Saitama Medical University, Saitama 350-0495, Japan; ‡‡ Department of Genome-based Drug Discovery, Graduate School of Biomedical Sciences, Nagasaki University, Nagasaki 852-8521, Japan; §§ iPSC-based Drug Discovery and Development Team, RIKEN BioResource Research Center (BRC), Kyoto 619-0238, Japan; ¶¶ Medical-risk Avoidance based on iPS Cells Team, RIKEN Center for Advanced Intelligence Project (AIP), Kyoto 606-8507, Japan; ‖‖ Department of Neurology, Graduate School of Medicine, University of Tokyo, Bunkyo, Tokyo 113-8655, Japan

**Keywords:** Alzheimer disease, induced pluripotent stem cell (iPS cell) (iPSC), amyloid-beta (AB), amyloid precursor protein (APP), lectin, beta-secretase 1 (BACE1), microarray, neurodegeneration, galectin 3–binding protein (GAL3BP), glycophosphatidylinositol-specific phospholipase D1 (GPLD1)

## Abstract

Alzheimer's disease (AD) is the most common type of dementia, and its pathogenesis is associated with accumulation of β-amyloid (Aβ) peptides. Aβ is produced from amyloid precursor protein (APP) that is sequentially cleaved by β- and γ-secretases. Therefore, APP processing has been a target in therapeutic strategies for managing AD; however, no effective treatment of AD patients is currently available. Here, to identify endogenous factors that modulate Aβ production, we performed a gene microarray–based transcriptome analysis of neuronal cells derived from human induced pluripotent stem cells, because Aβ production in these cells changes during neuronal differentiation. We found that expression of the glycophosphatidylinositol-specific phospholipase D1 (*GPLD1*) gene is associated with these changes in Aβ production. GPLD1 overexpression in HEK293 cells increased the secretion of galectin 3–binding protein (GAL3BP), which suppressed Aβ production in an AD model, neuroglioma H4 cells. Mechanistically, GAL3BP suppressed Aβ production by directly interacting with APP and thereby inhibiting APP processing by β-secretase. Furthermore, we show that cells take up extracellularly added GAL3BP via endocytosis and that GAL3BP is localized in close proximity to APP in endosomes where amyloidogenic APP processing takes place. Taken together, our results indicate that GAL3BP may be a suitable target of AD-modifying drugs in future therapeutic strategies for managing AD.

## Introduction

Alzheimer's disease (AD)^2^ is a progressive neurodegenerative disease with dementia and is characterized by the presence of amyloid plaques and neurofibrillary tangles in a wide range of cerebral cortex and limbic regions. The amyloid plaque is formed from β-amyloid peptides (Aβ) consisting of ∼40 amino acids (1, 2), and its accumulation is considered an early pathological hallmark. Thus, abnormal production and accumulation of Aβ are involved in the pathogenesis of AD (3, 4).

Aβ is produced from the amyloid precursor protein (APP) via sequential cleavages by β- and γ-secretases (5). β-Site amyloid precursor protein-cleaving enzyme 1 (BACE1) is the enzyme responsible for β-secretase activity and cleaves the N-terminal side of the Aβ region in APP (β-cut) to produce a soluble form of APP (sAPPβ) and the APP C-terminal fragment (APP-βCTF). A membrane protein complex consisting of presenilin, nicastrin, Aph-1, and Pen-2 is responsible for the γ-secretase activity that cleaves the C-terminal side of the Aβ region in the APP-βCTF (γ-cut), and the cleaved Aβ is secreted into the extracellular space. γ-Secretase cleavage occurs at several positions within the transmembrane region of the APP-CTFs, resulting in the production of a mixture of Aβs with varying length and aggregation properties, such as Aβ40 and Aβ42 (6–8). The longer Aβ, Aβ42, exhibits strong neurotoxicity and intrinsic self-assembly properties, promoting its accumulation in the extracellular amyloid plaques (9). There is also an alternative processing of APP, which is the cleavage by an α-secretase, resulting in the production of smaller C-terminal fragments, p3 (10). A large part of the N-terminal portion of APP is shed from the cell membrane after α-cleavage, and the fragment is called sAPPα. APP is shuttled to the plasma membrane from the *trans*-Golgi network, processed first by α-secretases and then by γ-secretases for the nonamyloidogenic pathway. On the other hand, for the amyloidogenic pathway, APP that escaped from α-cleavage is brought back from the cell surface to acidic early endosomes, where β- and γ-secretases sequentially cleave APP to produce amyloidogenic Aβ40 and Aβ42 (11–13).

Modulations in enzyme activities of β- and γ-secretases have been expected to be therapeutic targets in AD. However, recent clinical trials aiming at γ-secretase inhibition resulted in failure because of unwanted effects (14). This is thought to be due to the complex enzymatic action of the γ-secretase and its wide range of substrates, including essential signaling molecules, such as Notch and ErbB4, in addition to APP (15, 16). β-Secretase inhibitors have been considered more promising drugs in AD than those for γ-secretase because the phenotype of the BACE1 knockout mice is less pronounced than those of mice with knocked out components of the γ-secretase complex (17). However, β-secretase also has various endogenous substrates, such as NGR1, Jag1/2, and Sez6/Sez6L, and is involved in the processing of these proteins for their maturation or activation (18). Taken together, it is necessary to identify new target molecules and/or genes that can reduce Aβ production with no or fewer unwanted effects.

Induced pluripotent stem (iPS) cells differentiate to a variety of cells *in vitro* and, thus, reproduce the innate intracellular environment or components (19, 20). We previously found that production of both Aβ40 and Aβ42, as well as the ratio of Aβ40/42, are changed during neuronal differentiation of iPS cells (19). These findings strongly suggest the possibility that there are some factors that regulate β- and/or γ-cleavage of APP, and in this study, we searched for such factors that could be a therapeutic target in AD.

## Results

### Identification of GPLD1 as a candidate gene that modulates Aβ production

**Figure 1.**
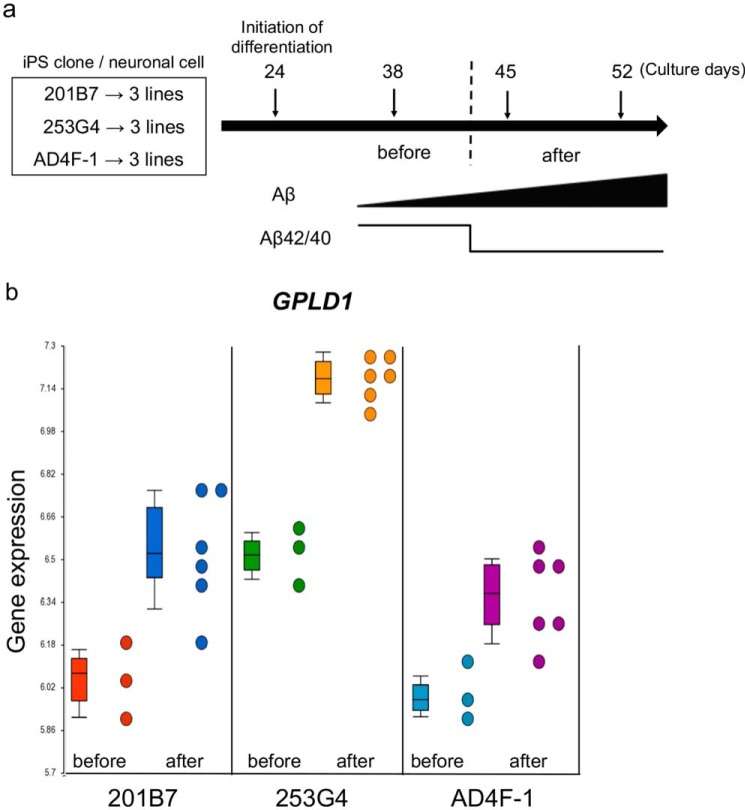
**Identification of *GPLD1* as a candidate gene related to changes in Aβ production during neuronal differentiation of iPS cells.** *a*, *schematic representation* of the experimental design. The iPS cell clones 201B7 and 253G4 were derived from healthy people with normal cognitive functions. The AD4F-1 clone was derived from a patient with sporadic AD. Three cultures derived from each of the iPS clones were subjected to neuronal differentiation and harvested for RNA preparation at days 38, 45, and 52. The amounts of secreted Aβ40 and Aβ42 increased during neuronal differentiation. The Aβ42/40 ratio was dramatically decreased at days 45 and 52 compared with that at day 38. We termed samples from day 38 as “before” and samples from days 45 and 52 as “after” according to the observed changes in the Aβ42/40 ratio. *b*, the GPLD1 expression levels in the three cultures derived from each of the original iPS cells (201B7, 253G4, and AD4F-1) during neuronal differentiation were compared before and after changes of the Aβ42/40 ratio. The average of the -fold increase in GPLD1 among the three lines is 1.42 (1.59-, 1.85-, and 1.29-fold for 201B7, 253G4, and AD4F-1, respectively). *Error bars*, S.D.

We previously reported that neuronal cells that are differentiated from human iPS (hiPS) cells express APP and secrete Aβ into the culture medium (19). When the expression of APP and the ratio Aβ42/40 of the secreted proteins were measured at days 38, 45, and 52 during differentiation, both APP expression and the secretion of Aβ40 and Aβ42 at days 45 and 52 were significantly increased compared with those at day 38 (19). Additionally, the expression of BACE1 was also increased at days 45 and 52 compared with day 38 (19). Interestingly, the ratio of Aβ42/40 was dramatically reduced at days 45 and 52 compared with that at day 38 (19). We hypothesized that transcriptional changes during the differentiation into neuronal cells may affect APP production and/or processing independently of the increased BACE1 expression. To test this hypothesis, we performed microarray analyses using three independent neuronal cell cultures that were differentiated from each of three hiPS cells, namely 201B7, 253G4, and AD4F-1 (nine cell lines in total; Fig. 1*a*). Total RNA was prepared at three differentiation time points (days 38, 45, and 52) and subjected to an Affymetrix Gene Chip Human Exon 1.0 ST array. The principal component analysis showed a unique pattern among the three groups of the hiPS cell lines 201B7 (*red*), 253G4 (*blue*), and AD4F-1 (*green*) (Fig. S1). Next, we examined genes showing common expressional changes among these three groups during the differentiation. In particular, we focused on genes differentially expressed before and after the drastic changes in APP expression, Aβ production, and the ratio of Aβ42/40 (*i.e.* comparison between samples from 38-day cultures and samples from 45- and 52-day cultures) using the Partek Genomics Suite software. A total of 316 genes showing an at least 1.3-fold expressional change with statistical significance (*p* < 0.05) were detected as candidates correlated with the changes in Aβ production and the Aβ42/40 ratio (Table S1). Among these genes, we particularly paid attention to *GPLD1* encoding glycophosphatidylinositol-specific phospholipase D1, which cleaves the inositol phosphate linkage in proteins modified with a GPI anchor (21, 22), because Aβ is produced from APP in lipid rafts where GPI-anchored proteins are associated (23, 24). Thus, we hypothesized that GPLD1 regulates intracellular trafficking and/or localization into lipid rafts of GPI-anchored proteins, and these changes may affect APP processing or, alternatively, that GPLD1 cleaves GPI-anchored proteins, and the resulting products may regulate Aβ production/accumulation in an autocrine or paracrine manner.

**Figure 2.**
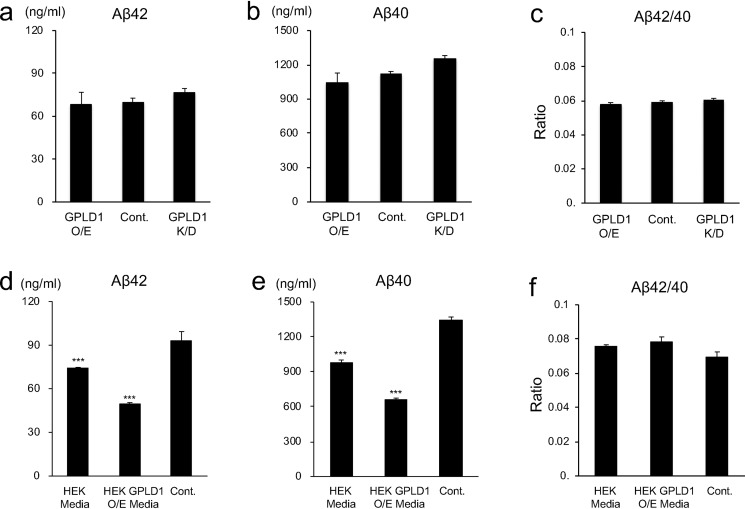
**GPLD1-dependent suppression of Aβ production.** *a–c*, GPLD1 expression vector (*GPLD1 O/E*) or GPLD1 shRNA (*GPLD1 K/D*) was transfected into H4-APPsw cells, and the amounts of secreted Aβ40 and Aβ42, as well as the ratio of Aβ42/40, were measured in the conditioned media using ELISAs (*n* = 3, mean ± S.D. (*error bars*)). *p* > 0.05 *versus* control by one-way ANOVA with Tukey's post-hoc test. *d–f*, GPLD1 was transiently expressed in HEK293 cells. The conditioned media from HEK293 cells without (*HEK Media*) or with (*HEK Media GPLD1 O/E*) GPLD1 expression were added to H4-APPsw cells, and these cells were cultured for 24 h. Cell cultures with fresh media were used as controls. The amounts of the secreted Aβ40 and Aβ42, as well as the ratio of Aβ42/40, were measured in the conditioned media using ELISAs (*n* = 3, mean ± S.D.). ***, *p* < 0.001 *versus* control by one-way ANOVA with Tukey's post-hoc test.

Gene expression data showed a significant up-regulation of *GPLD1* in all three hiPS cell–derived neuronal cells (Fig. 1*b*). Thus, we examined whether overexpression or knockdown of *GPLD1* affects Aβ production in the human neuroglioma H4 cell that stably expresses APP with the Swedish mutation (H4-APPsw) (25). However, we did not observe significant changes in the production of either Aβ42 or Aβ40 after overexpression or knockdown via RNAi (Fig. 2 (*a* and *b*) and Fig. S2). The ratio of Aβ42/40 was also unchanged (Fig. 2*c*). We hypothesized that some factors involved in the GPLD1-induced modulation of the Aβ production are not sufficiently expressed in the H4 cell line. HEK293 cells have often been used to study APP processing and Aβ production (26) and also are known to express a wide range of membrane-bound and secreted proteins (27). Therefore, we transfected *GPLD1* into HEK293 cells, collected the conditioned media, and added these HEK293-conditioned media to H4-APPsw cell cultures to investigate changes in Aβ production. Surprisingly, productions of both Aβ40 and Aβ42 were significantly reduced by replacing the H4-APPsw media with the HEK293-conditioned media with or even without GPLD1 expression (Fig. 2, *d* and *e*). The effect on the reduction of Aβ production was more efficient with GPLD1 overexpression, although the ratio of Aβ42/40 remained unchanged in all conditions (Fig. 2*f*). We also transfected GPLD1 into HEK293 cells that stably expressed APPsw (HEK293-APPsw cells) and confirmed that the production of Aβ40 and Aβ42 was reduced significantly in HEK293-APPsw–conditioned media (Fig. S3).

### Identification of GAL3BP as an autocrine/paracrine factor suppressing Aβ production

**Figure 3.**
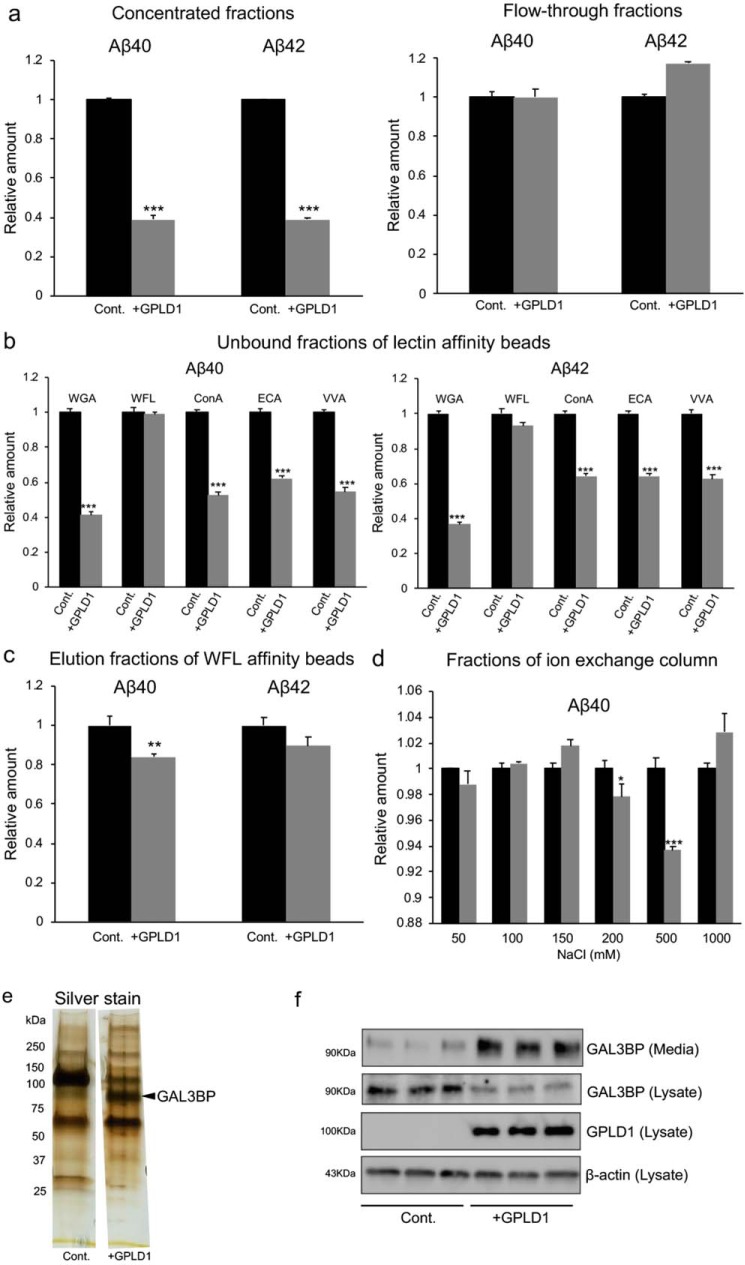
**Identification of GAL3BP as a factor suppressing Aβ production.** *a*, filtration. Conditioned media from HEK293 cells (*Cont*.) and HEK293 cells expressing GPLD1 (+*GPLD1*) were subjected to Amicon Ultra 30-kDa centrifugal filter units. H4-APPsw cells were incubated with the concentrated and flow-through fractions to test their suppressive activity on the production of Aβ40 and Aβ42. *b*, lectin affinity beads. The concentrated fractions of *a* were subjected to lectin affinity beads (WGA-, WFL-, ConA-, ECA-, and VVA-beads), and the unbound fractions were tested for their inhibitory activity on the production of Aβ40 and Aβ42. *c*, WFL-affinity beads. Materials bound to WFL-beads were eluted with GalNAc, and the eluted fractions were tested for their suppressive activity on the production of Aβ40 and Aβ42. *d*, ion-exchange columns. The eluted fractions from WFL-beads were subjected to ion-exchange columns. The bound materials were eluted with NaCl (50, 100, 150, 200, 500, and 1000 mm), and the elution fractions were tested for their suppressive activity on the production of Aβ40. *e*, silver staining of the purified fractions. Eluted fractions from the ion-exchange column with 0.5 m NaCl were subjected to SDS-PAGE and silver-stained. A band around 90 kDa in the purified fractions (*arrowhead*) was subjected to MS analysis to identify the protein. *f*, expression of GAL3BP in HEK293 cells. The cDNA encoding GPLD1 was transfected into HEK293 cells. The amounts of GAL3BP proteins in the cell culture media and in the cell lysates and that of GPLD1 in the cell lysates were analyzed by Western blotting. β-Actin was used as a loading control. *a–d*, the values in the graphs are presented relative to the corresponding controls (*n* = 3, mean ± S.D. (*error bars*)). *, *p* < 0.05; **, *p* < 0.01; ***, *p* < 0.001 *versus* control by one-way ANOVA with Tukey's post-hoc test.

To identify the factors that can inhibit Aβ production in the conditioned media from GPLD1-expressing HEK293 cells, we first concentrated the conditioned media using Amicon Ultra centrifugal filters with a 30-kDa molecular mass cutoff. The concentrated but not the flow-through fractions decreased the production of both Aβ40 and Aβ42 (Fig. 3*a*). Because extracellular domains of membrane-bound or secreted proteins are often modified with glycans, we thought that lectin affinity beads could capture the suppressive factors. We therefore applied the concentrated fractions to several beads conjugated to lectins, namely wheat germ agglutinin (WGA), *Wisteria floribunda* agglutinin (WFL), concanavalin A (ConA), *Erythrina cristagalli* lectin (ECA), and *Vicia villosa* lectin (VVA), to examine whether any of them can capture the suppressive factors. The suppressive activities were detected in the flow-through fractions of WGA-, ConA-, ECA-, and VVA-beads, but not in the flow-through fractions of WFL-beads (Fig. 3*b*). The eluted fraction from WFL-beads with GalNAc suppressed the Aβ production in H4-APPsw cells (Fig. 3*c*). We applied the WFL elution to ion-exchange columns, and the bound proteins were recovered with stepwise NaCl elution. The fraction eluted with 0.5 m NaCl exhibited suppressive activities (Fig. 3*d*). Silver staining showed a unique band around the molecular mass of 90 kDa in the samples purified from the conditioned media of GPLD1-expressing HEK293 cells (Fig. 3*e*). Mass spectrometry analysis identified that this band corresponds to the human GAL3BP (Fig. 3*e* and Table S2). Western blot analysis showed that the amount of GAL3BP protein decreased in cell lysates and increased in the culture media following the overexpression of GPLD1 (Fig. 3*f*). RT-PCR analysis showed no obvious changes in the expression of GAL3BP mRNA in HEK293 cells with or without GPLD1 overexpression (Fig. S4). These data suggest that HEK293 cells endogenously secrete GAL3BP and that overexpression of GPLD1 increases the amount of secreted GAL3BP (Fig. 3*f*). We also confirmed that H4-APPsw cells do not express detectable amounts of GAL3BP in either cell lysates or culture media (Fig. S5), which may explain the absence of detectable change in Aβ production in H4-APPsw cells despite GPLD1 overexpression (Fig. 2).

Although the reasons for the relatively weak inhibitory activities in the fractions purified later compared with the concentrated fractions are unknown, the inhibitory activities may have been partially lost during purification. Moreover, the concentrated fractions may possibly contain factors other than GAL3BP with protective effects on APP processing. Another possible explanation for its weak activity in WFL elution fractions (Fig. 3*c*) could be the presence of GalNAc, an ingredient of WFL elution buffer, which inhibits the activity of GAL3BP on Aβ production in H4-APPsw cells (Fig. S6).

**Figure 4.**
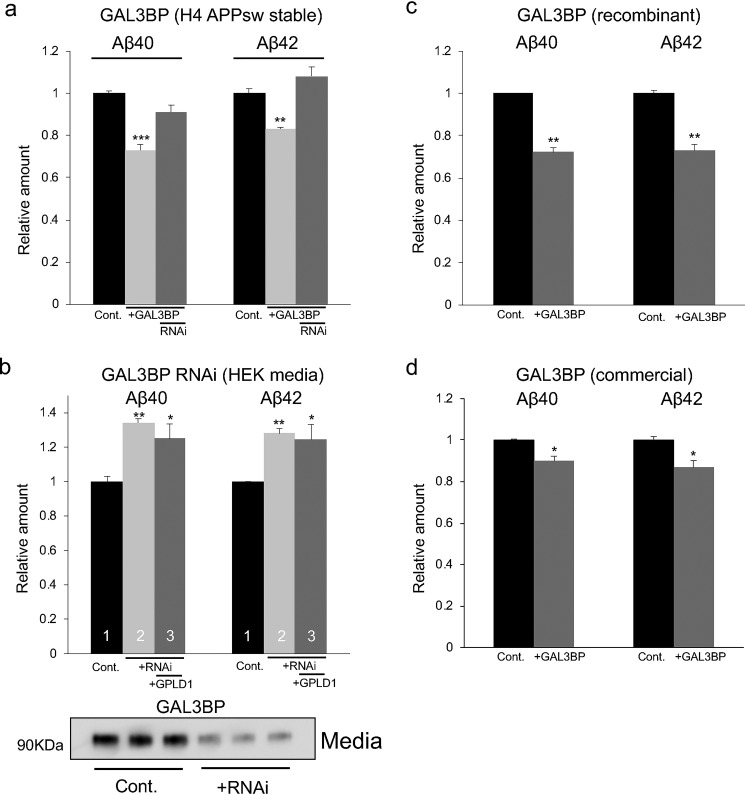
**GAL3BP-induced inhibition of Aβ production.** *a*, the amounts of Aβ40 and Aβ42 in the conditioned media from H4-APPsw cells (*Cont*.) and H4-APPsw cells overexpressing GAL3BP (+*GAL3BP*) were measured using ELISAs. *b*, conditioned media were prepared from control HEK293 cells (*Cont*., *lane 1*), HEK293 cells with GAL3BP knockdown via RNAi (+*RNAi*, *lane 2*), and HEK293 cells with GAL3BP knockdown and GPLD1 overexpression (+*RNAi*, +*GPLD1*, *lane 3*) and subsequently added to H4-APPsw cells to test for their suppressive activity on Aβ production. Western blot analysis confirmed the efficiency of RNAi for the amount of GAL3BP expression in the culture media (*bottom*). *c*, recombinant GAL3BP (5 μg/ml) prepared from HEK293 cells was added to H4-APPsw cell cultures, and the amounts of Aβ40 and Aβ42 in the media were determined. *d*, commercially available recombinant GAL3BP was added to H4-APPsw cell culture, and the amounts of Aβ40 and Aβ42 in the media were measured. *a–d*, the values in this graph are presented relative to the corresponding controls (*n* = 3, mean ± S.D. (*error bars*)). If error bars representing S.D. values are not visible, they are too small to be displayed. *, *p* < 0.05; **, *p* < 0.01; ***, *p* < 0.001 *versus* control by one-way ANOVA with Tukey's post-hoc test.

To confirm whether GAL3BP possesses the suppressive activity on Aβ production, we first transfected *GAL3BP* cDNA in H4-APPsw cells. Overexpression of GAL3BP inhibited the production of both Aβ40 and Aβ42 (Fig. 4*a*). Next, we knocked down GAL3BP in HEK293 cells via RNAi and added these conditioned media to H4-APPsw cell culture. Fig. 4*b* shows that knockdown of endogenous GAL3BP in HEK293 cells increased the production of both Aβ40 and Aβ42, indicating that the suppressive activity of the Aβ production was attenuated. Furthermore, when GAL3BP was knocked down in HEK293 cells, overexpression of GPLD1 had no effect on Aβ production (Fig. 4*b* and Fig. S7), suggesting that GAL3BP acts as a downstream factor of GPLD1. Finally, the addition of recombinant GAL3BP protein into H4-APPsw cell culture media decreased the production of both Aβ40 and Aβ42 (Fig. 4*c*). Because recombinant GAL3BP preparations contain other contaminating proteins, we also used commercially available GAL3BP purified from a mouse myeloma cell line (Fig. S8). The commercially available GAL3BP also exhibited a similar inhibitory activity on Aβ production without changing cell viability. (Fig. 4*d* and Fig. S9).

We also tested the effects of GAL3BP on Aβ production in H4 cells that stably express WT APP (H4-APPwt). The results show around 30% suppression of both Aβ40 and Aβ42 (Fig. S10). The suppressive activity of the Aβ production by GAL3BP was also observed in human neuroblastoma SH-SY5Y cells (Fig. S11). These data indicate that GAL3BP can modulate Aβ production via autocrine/paracrine-like actions.

### Mechanism of GAL3BP-induced suppression of Aβ production

**Figure 5.**
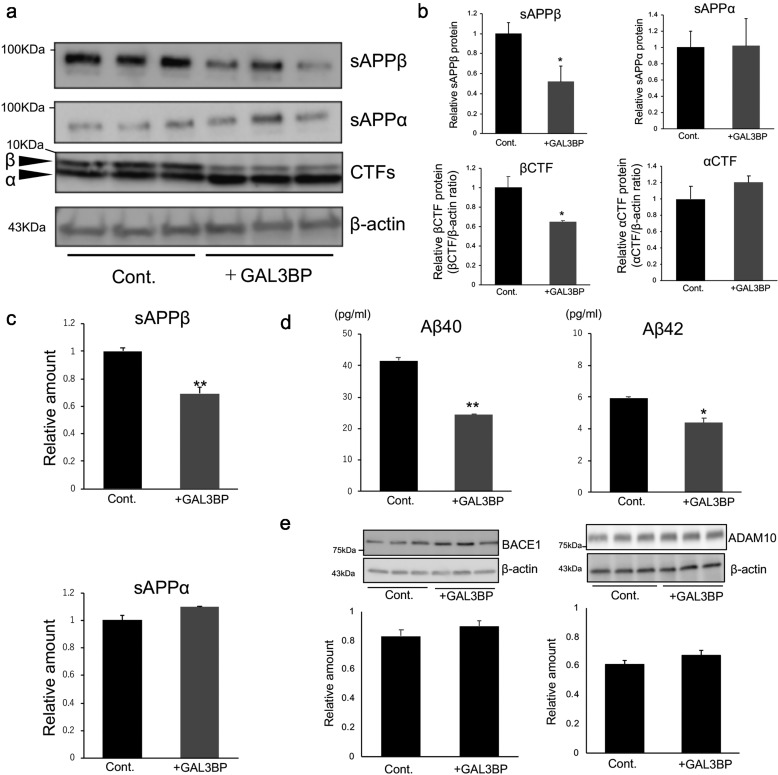
**APP processing by GAL3BP-treatments.** H4-APPsw cells were treated with (+*GAL3BP*) or without (*Cont*.) the commercially available GAL3BP. *a* and *b*, sAPPα and sAPPβ proteins in the culture media and CTFs in the cell lysates were detected with Western blot analysis. Blots with β-actin were used as a loading control. The relative sAPPβ and sAPPα in the culture media from GAL3BP-treated cells were normalized to the control samples. The relative CTFs were normalized to the corresponding β-actin levels. *c*, the amounts of sAPPβ and sAPPα in the conditioned media and βCTF in the cell lysates were measured using ELISAs. *d*, the amounts of Aβ40 and Aβ42 in the lysate were measured using ELISAs. *e*, Western blot analysis of BACE1 and ADAM10 in the lysates of H4-APPsw cells treated with or without GAL3BP. Blots with β-actin were used as a loading control. The relative BACE1 and ADAM10 values are normalized to the corresponding β-actin levels. *p* > 0.05 *versus* control by one-way ANOVA with Tukey's post-hoc test. *b–e*, the controls were vehicle controls, and the values in this graph are presented relative to each control (*n* = 3, mean ± S.D. (*error bars*)). *, *p* < 0.05; ***, *p* < 0.001 *versus* control by one-way ANOVA with Tukey's post-hoc test.

The transmembrane protease BACE1 cleaves APP and produces a soluble extracellular fragment (*i.e.* sAPPβ) and APP-βCTF, which in turn is processed by γ-secretase to Aβ. Alternatively, APP is processed by the α-secretase, a disintegrin and metalloproteinase domain-containing protein 10 (ADAM10), which produces nonamyloidogenic sAPPα and p3 fragments (11, 12). Thus, suppression of Aβ production by GAL3BP may be associated with changes in expression and/or activities of BACE1 or ADAM10. We therefore examined the sAPPα and sAPPβ levels in conditioned media of H4-APPsw cells with or without the addition of commercially available GAL3BP. Western blotting and ELISA show that the secretion of sAPPβ was reduced in the presence of GAL3BP, whereas the sAPPα levels were not changed (Fig. 5, *a–c*). In addition, the amount of intracellular βCTF significantly reduced after treatment with commercially available GAL3BP (Fig. 5, *a–c*). ELISA indicated no obvious intracellular accumulation of Aβ after GAL3BP treatments (Fig. 5*d*). The expression levels of BACE1 and ADAM10 were not altered by the addition of GAL3BP (Fig. 5*e*). These data indicate that GAL3BP affects the amyloidogenic APP processing pathway, possibly via inhibition of BACE1 activity. Thus, we tested whether GAL3BP modulates the enzyme activity of BACE1. BACE1 activity was measured in the presence of the purified recombinant GAL3BP fractions *in vitro*, and the results demonstrate the inhibitory effect of GAL3BP on BACE1 activity (Fig. S12*a*). A similar inhibitory effect on BACE1 activity was observed with the commercially available GAL3BP (Fig. S12, *b* and *c*).

**Figure 6.**
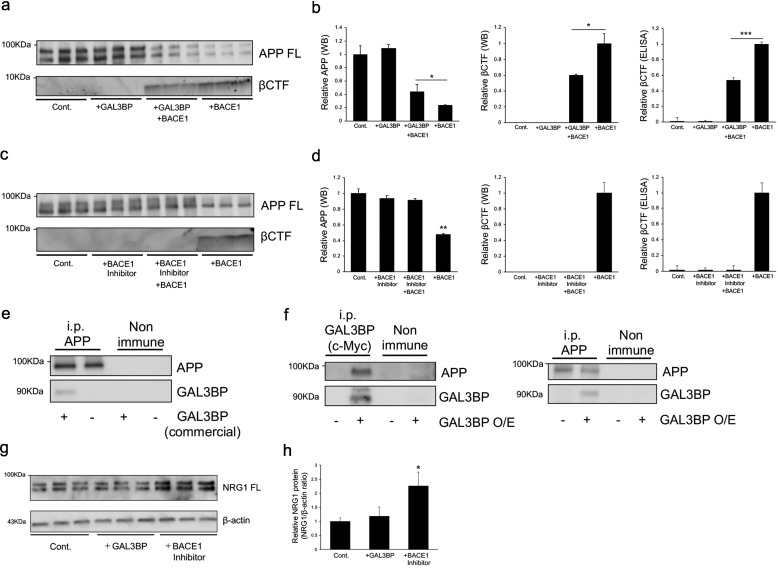
**Interaction of APP and GAL3BP.** *a–d*, inhibitory effects of GAL3BP on the processing of immunoprecipitated APP by BACE1. Immunoprecipitated APP from H4-APPsw cells were treated with BACE1 (2.5 unit/ml) in the presence or absence of the commercially available GAL3BP (10 μg/ml) (*a* and *b*). Immunoprecipitated APP was also treated with BACE1 (2.5 unit/ml) and BACE1 inhibitor (50 nm) (*c* and *d*). The samples were then analyzed with Western blotting (*a* and *c*) and ELISAs to detect full-length (*FL*) APP and βCTF. The relative values for full-length APP and βCTF were normalized (*b* and *d*). The whole gel images are shown in Fig. S13. The values in this graph are presented relative to each other and the corresponding controls (*n* = 3, mean ± S.D. (*error bars*)). *, *p* < 0.05; **, *p* < 0.01; ***, *p* < 0.001 *versus* control by one-way ANOVA with Tukey's post-hoc test. *e*, direct interaction of APP and GAL3BP. The immunoprecipitated APP proteins from H4-APPwt cells were incubated with the commercially available GAL3BP, and then the bound materials were analyzed with Western blotting using antibodies against APP and GAL3BP. Nonimmunoglobulin was used as a control. *f*, co-immunoprecipitation of GAL3BP and APP. The cDNA encoding GAL3BP with the c-Myc tag was transfected into H4-APPsw cells. The cell lysates were subjected to immunoprecipitation with anti-APP or anti-c-Myc antibodies, and then immunoprecipitated materials were analyzed with Western blotting. The blots were probed with anti-APP (*top panels*) and anti-GAL3BP (*bottom panels*) antibodies. Nonimmunoglobulin was used as a control. *g* and *h*, effect of GAL3BP on NRG1 processing. H4-APPsw cells were treated with the commercially available GAL3BP or the BACE1 inhibitor, and the cell lysates were analyzed with Western blotting using the antibody to NRG1. Blots with β-actin were used as a loading control. The values in this graph are presented relative to each other and the corresponding controls (*n* = 3, mean ± S.D.). *, *p* < 0.05 *versus* control by one-way ANOVA with Tukey's post-hoc test.

A possible mechanism for the inhibition of BACE1 activity by GAL3BP could be direct interaction; however, thus far, we could not detect any direct interaction between them. Instead, we hypothesized that GAL3BP may bind to the substrates of BACE1 and thus protect BACE1-dependent processing. However, the identity of the substrate used in the β-secretase assay kit is proprietary information. Thus, we performed an *in vitro* BACE1 assay using immunoprecipitated APP proteins as the substrate. As shown in Fig. 6 (*a* and *b*), the amount of full-length immunoprecipitated APP proteins decreased in the presence of BACE1, and the addition of the commercially available GAL3BP prevented such decreases. Accordingly, the production of APP-βCTF was suppressed in the presence of GAL3BP (Fig. 6, *a* and *b*). The aforementioned decreases of the full-length APP proteins and protective effects on the production of APP-βCTF were also observed when BACE1 inhibitor was used (Fig. 6, *c* and *d*), supporting the specificity of GAL3BP on the inhibition of BACE1. Subsequently, we incubated the immunoprecipitated APP proteins with commercially available GAL3BP to observe direct protein-protein interaction. The results showed that GAL3BP bound to immunoprecipitated APP–anti-APP antibody/protein A–beads, but not to the nonimmunoglobulin/protein A–beads (Fig. 6*e*). Moreover, GAL3BP and APP were co-immunoprecipitated from the GAL3BP-overexpressing H4-APPsw cells (Fig. 6*f*). These data suggest an interaction between GAL3BP and APP, which suppresses BACE1-dependent APP processing and consequent Aβ production, possibly via physical protection of BACE1 accessibility to the β-cut site. Detailed interaction sites should be investigated in the future. The addition of the commercially available GAL3BP to the H4-APPsw culture media did not affect the processing of NRG1, another substrate of BACE1, whereas the addition of BACE1 inhibitor, which directly binds to the active site of BACE1, increased the amount of full-length NRG1 (Fig. 6, *g* and *h*). These data may suggest a selective suppression of APP processing by GAL3BP through direct interaction.

**Figure 7.**
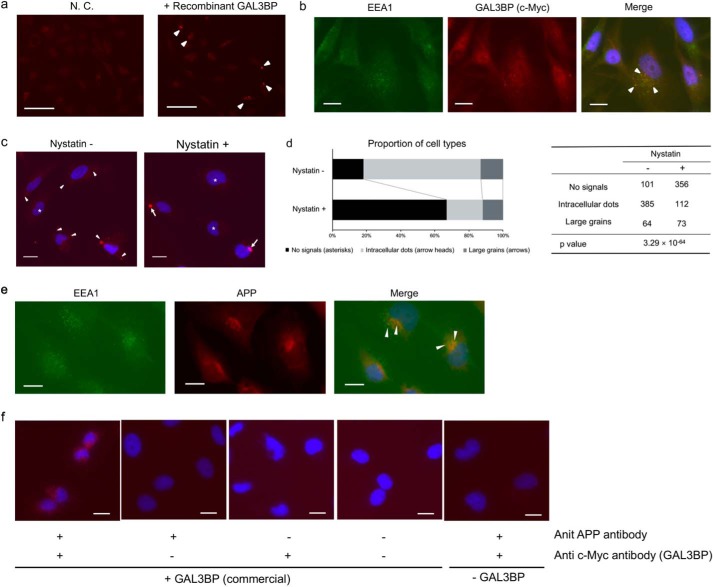
**Immunofluorescence analysis of the intracellular location of extracellularly added GAL3BP.** *a*, H4-APPsw cells were incubated with extracellularly applied recombinant GAL3BP (5 μg/ml) for 24 h and then stained with anti-GAL3BP (c-Myc) antibody. *Arrowheads*, intracellular signals of recombinant GAL3BP. Vehicle treatment was used as negative control (*N.C.*). *Scale bars*, 50 μm. *b*, H4-APPsw cells treated with GAL3BP were co-stained for GAL3BP (c-Myc), the early endosome marker EEA1, and nuclei (*DAPI*). *Arrowheads*, co-localization of intracellular signals of recombinant GAL3BP and EEA1. *Scale bars*, 20 μm. *c*, H4-APPsw cells were pre-incubated with or without nystatin (50 μg/ml) for 1 h and then incubated with recombinant GAL3BP for 3 h. The cells were stained for GAL3BP (*red*) and DAPI (*blue*). *Asterisks*, cells with no signal for intracellular GAL3BP. *Arrowheads*, intracellular dotlike signals of recombinant GAL3BP. *Arrows*, large grainlike signals for GAL3BP. *Scale bars*, 20 μm. *d*, quantification of intracellular signals for GAL3BP. Cells were classified into three groups: no intracellular signals, intracellular dotlike signals, and large grainlike signals (corresponding to *asterisks*, *arrowheads*, and *arrows* in *c*, respectively). More than 500 cells were counted in randomly taken fields and statistically analyzed using the χ^2^ test. *e* and *f*, H4-APPsw cells were treated with the recombinant GAL3BP (c-Myc–tagged). The cells were co-stained for APP, EEA1, and nuclei (DAPI) (*e*). *Arrowheads*, co-localization of intracellular signals of APP and EEA1 (*e*). The cells were also subjected to a PLA using antibodies to APP and c-Myc (*f*). *Red signals* indicate the co-localization of intracellular signals of APP and recombinant GAL3BP (*f*). *Scale bars*, 20 μm.

Finally, we examined whether the extracellularly added GAL3BP can be taken up by the cells. H4-APPsw cells treated with or without the recombinant GAL3BP were immunostained for this protein. We detected intracellular immunosignals for GAL3BP in cells treated with GAL3BP (Fig. 7*a*). These intracellular GAL3BP signals were merged with the signals for early endosome antigen 1 (EEA1), a marker for early endosomes (Fig. 7*b*, *arrowheads*). These intracellular signals for GAL3BP were significantly reduced when the cells were treated with nystatin, an endocytosis inhibitor (Fig. 7, *c* and *d*). Moreover, immunofluorescence analysis showed colocalization of APP with EEA1 in H4-APPsw cells; the results of proximity ligation assay (PLA) showed localization of APP and exogenously added recombinant GAL3BP in close proximity to each other (Fig. 7, *e* and *f*). Different subcellular locations of BACE1 (early endosome) and ADAM10 (*trans*-Golgi network and plasma membrane) may also affect the selective inhibition of β-cut, but not α-cut (28). In summary, our data demonstrate that GAL3BP suppresses the pathogenic production of Aβ via modulating the β-cut of APP.

## Discussion

In this study, we found that GAL3BP directly suppresses the β-cut of APP and reduces Aβ production. BACE1 has been a target for therapeutic drug development in AD for many years. Aβ42 shows a higher self-aggregation propensity than Aβ40 and is thus regarded to have a more pathogenic contribution (13). We previously reported that hiPS cell–derived neuronal cells change the levels of Aβ production and the ratio of Aβ42/40 in the course of neuronal cell differentiation (19). Therefore, we expected that this system could be a screening model allowing us to identify molecules involved in APP processing and Aβ production. Among the 316 genes that were detected by the transcriptome analysis as candidates correlating well with the changes in Aβ production and the Aβ42/40 ratio, we focused on GPLD1 in this study. GPLD1 is a GPI-cleaving enzyme, and GPI-anchored proteins accumulate in lipid rafts (29). A raft is a microdomain of the membrane where APP processing and Aβ production occur. Many studies have indicated a relationship between Aβ production in the rafts and AD pathogenesis (11, 12, 30). We hypothesized that GPLD1 produces autocrine/paracrine factors. The models in our present study suggest that, dependent on the GPLD1 expression, GAL3BP is secreted into the cell culture medium and incorporated into the endosomes and subsequently modulates APP processing, which results in reduced Aβ production. Because our data indicate that GAL3BP did not change the Aβ42/40 ratio, other factors among the 316 genes identified by our transcriptome analysis are probably involved in this process. Moreover, it is still possible that proteins other than GPLD1 and GAL3BP also suppress Aβ production. Although GPLD1/GAL3BP may be insufficient to completely block the BACE1-dependent Aβ production, we propose that our transcriptome data of hiPS cells during neuronal differentiation provide information to identify additional modulators that affect APP processing and Aβ production.

It has been reported that GPLD1 is released by cells and can cleave GPI-anchored proteins in a para- and endocrine fashion (31). In the brains of AD patients, the expression levels of GPLD1 were not changed, compared with those in healthy human brains, but interestingly, GPLD1 in the cerebrospinal fluid of AD patients shows a lower molecular weight compared with that of healthy individuals (29), suggesting abnormalities in post-translational modifications such as glycosylation. Generally, glycosylation regulates the function and stability of proteins, so it is possible that GPLD1 activity and/or stability may be changed in AD patients.

The mechanism of how GPLD1 promotes the release of GAL3BP is not known. Because GAL3BP is unlikely to be a GPI-anchored protein, it may not be a direct target of GPLD1. One possibility is that overexpression of GPLD1 might have induced the cleavage of some GPI-anchored proteins and consequently affected the raft structure and function as well as endocytic mechanisms. Such cellular responses may have increased the secretion of GAL3BP. GAL3BP was originally identified as a binding protein to a human macrophage-associated lectin known as galectin 3 (32). Interestingly, the expression of galectin 3 is elevated in the serum of AD patients, suggesting an association between AD pathology and the galectin 3–GAL3BP axis (33, 34). Because galectin 3 controls retention and endocytosis of cell surface proteins (35), it is possible that abnormal expression and/or regulation of galectin 3 may be associated with AD, and GAL3BP may suppress such pathogenic effects of galectin 3 through direct interaction. Alternatively, galectin 3 may deprive GAL3BP of its critical function to modulate APP processing. Detailed studies examining GPLD1 and GAL3BP in the human AD brain or in AD animal models would be of interest.

We found that GAL3BP inhibited Aβ production by about 30%. A legitimate question is whether only 30% inhibition can lead to therapeutic effects. Given that BACE1 has several endogenous substrates other than APP and that mice with loss of BACE1 activity present abnormal behavior (36–38) and axonal disorganization (39), a partial suppression of BACE1 activity is desirable for long-term therapeutic agents in AD. In fact, the rare A673T mutation in *APP*, suppressing the β-cut and Aβ production by as much as 40%, is protective regarding AD onset (40–42). In addition, GAL3BP suppresses Aβ production via direct interaction with APP, ensuring target specificity as compared with other BACE1 inhibitors. Taken together, a GAL3BP-mediated moderate suppression of Aβ production is expected to have sufficient therapeutic effects. Even a partial inhibition would generate a large benefit in terms of a delayed AD onset, considering its long course (*i.e.* the aggregation of Aβ and the formation of amyloid plaques).

A major obstacle for AD treatment is the timing of intervention. Therapies usually start when the patients show apparent cognitive deficits, but at this time point, Aβ is already accumulated, and the pathological cascade has started (43). GAL3BP is physiologically present in human serum (44, 45). If a correlation between GAL3BP levels in serum or cerebrospinal fluid and AD is established, GAL3BP may not only be a druggable molecule to reduce the AD risk, but it may also serve as a relevant biomarker for the AD pathogenesis. On the other hand, it is known that elevated serum levels of GAL3BP (>10 μg/ml) are associated with various forms of cancer (45) and venous thrombosis (46). Given that BACE1-dependent APP processing is already inhibited by GAL3BP within its physiological range (5–10 μg/ml in healthy controls) (45), overexpression of GAL3BP would not be necessary when considering therapeutic applications. Rather, it would be necessary to ensure that the GAL3BP level does not fall below its physiological threshold to prevent excessive Aβ production. In summary, we propose that our system combining transcriptome analyses of hiPS cells and biochemical and functional assays using AD model cells can be useful to find new target molecules for disease-modifying drugs in AD.

## Experimental procedures

### hiPS cell lines and microarray analysis

Cultures of hiPS cells and their differentiation into neuronal cells were described previously (19). Total RNA was obtained from neuronal cells differentiated from hiPS cells using TRIzol reagent (Invitrogen) Expression profiles of mRNA were obtained using GeneChip® Human Exon 1.0 ST Human Gene Expression Affymetrix arrays according to the manufacturer's instructions. Briefly, biotinylated cRNA was prepared from 250 ng of total RNA according to the standard protocol for the Affymetrix 3′ IVT Express Plus Reagent Kit. Following fragmentation, cRNA was hybridized for 16 h at 45 °C to Affymetrix Primeview Human arrays. The GeneChips were scanned using a GeneChip Scanner 3000 7G (Affymetrix, Santa Clara, CA). The CEL files were imported into Partek software version 6.5 (Partek Inc., St. Louis, MO) and normalized using robust multiarray normalization. Contrasts of interest were analyzed using a two-way analysis of variance (ANOVA). Using Partek Genomic Suite^TM^, a list of differentially expressed mRNAs was obtained for multiple-hypothesis testing using a false discovery rate of <0.3 and an adjusted *p* ≤ 0.05 with an at least 1.3-fold change.

### Cell culture

H4-APPsw cells that stably express the APP with Swedish mutation (47) exhibit increases in Aβ production due to increases in the β-cut of APP. H4-APPwt cells stably express the WT APP. Such cells were cultured in Dulbecco's modified Eagle's medium (DMEM; high glucose) with l-glutamine, phenol red, and sodium pyruvate (WAKO Pure Chemical Industries Ltd., Osaka, Japan) supplemented with fetal bovine serum (10%), penicillin (100 units/ml), streptomycin (100 μg/ml), and hygromycin (150 μg/ml). Cultures were maintained at 37 °C in a humidified 5% CO_2_ atmosphere. HEK293 cells were cultured in DMEM supplemented with 10% fetal bovine serum and penicillin/streptomycin. For cell lysis, the cells were washed with PBS and solubilized with a buffer containing 50 mm Tris-Cl, pH 7.4, 150 mm NaCl (TBS), 1% Triton X-100, and a protease inhibitor mixture (Nakalai Tesque, Kyoto, Japan). The samples were centrifuged at 14,000 rpm for 10 min, and the supernatants were used as cell lysates. Effectene transfection reagent (Qiagen, Hilden, Germany) was used for transfection of the plasmids encoding *GPLD1*, *GAL3BP*, *and APP* according to the manufacturer's instructions. We used G418 (100 μg/ml) for selection of cells stably expressing transfected cDNA. For the treatment of H4-APPsw cells with HEK293 cell–conditioned medium, HEK293 cells that had been transfected with or without GPLD1 were cultured for 24 h. The conditioned media were recovered and centrifuged at 4,000 rpm for 10 min. The supernatants were added to H4-APPsw cells that were cultured on a 6-well plate for an additional 24 h.

### Plasmid constructions

#### 

##### GPLD1

The cDNA encoding human *GPLD1* was cloned from HEK293 cells by RT-PCR using the TAAGAATTCACCATGTCTGCTTTCAGGTTGTGGC and TTTCTCGAGCTACTTGTCGTCATCGTCTTTGTAGTCTCAATCTGAGCCAAGGCTATAGACGTG primer pair. The PCR product was inserted into the EcoRI and XhoI sites of the pcDNA3.1 vector. GPLD1 was expressed as a fusion protein with a FLAG tag at the C terminus.

##### GAL3BP

The cDNA encoding human *GAL3BP* was cloned from HEK293 cells by RT-PCR using the ATCATGACCCCTCCGAGGCTCTTCTG and TTTTCTAGACTAGTCCACACCTGAGGAGTTGG primer pair. The PCR product was inserted into the EcoRV and XbaI site of the pcDNA3.1 that contains the signal sequence of Gaussia luciferase and His/Myc tags at the 5′ side (AATTAGCCACCATGGGAGTCAAAGTTCTGTTTGCCCTGATCTGCATCGCTGTGGCCGAGGCCAAGCCCACCCATCATCACCATCACCATGAACAAAAACTCATCTCAGAAGAGGATCTGAGGAATTCGAT) His/Myc vector (48). GAL3BP was expressed as a fusion protein with a signal sequence and His/Myc tag at the N terminus.

##### APP

The cDNA encoding human *APP* was cloned from HEK293 cells by RT-PCR using the TAAAAGCTTACCATGCTGCCCGGTTTGGCAC and AATCTAGACTAGTTCTGCATCTGCTCAAAGA primer pair. The PCR product was inserted into the HindIII and Xba sites of the pcDNA3.1 vector. For the generation of APP with the Swedish mutations (KM670/671NL), direct mutagenesis was performed using the TCATGTCGGAATTCTGCATCCAGATTCACTTCAGAGATCTCCTCCG primer.

### RNAi

The validated Stealth RNAi^TM^ (Invitrogen) was used for knockdown of GPLD1 (Oligo ID HSS142252, HSS142253, and HSS142254) and GAL3BP (Oligo ID HSS 180671, HSS180672, and HSS180673). Stealth RNAi^TM^ transfection was carried out using Lipofectamine2000 (Thermo Fisher Scientific).

### Western blot analysis

Protein samples were separated by SDS-PAGE and transferred to an Immobilon polyvinylidene difluoride membrane (Millipore, Burlington, MA) and nitrocellulose membrane (Amersham Biosciences, Buckinghamshire, UK). The membrane was blocked with 5% (w/v) skim milk in TBS-T (33 mm Tris buffer, pH 7.6, containing 100 mm NaCl and 0.1% Tween 20) or 1% BSA in PBS and then incubated with primary antibodies at 4 °C overnight. After washing with TBS-T, the membranes were further incubated with horseradish peroxidase–conjugated secondary antibodies in TBS-T. The membranes were washed with TBS-T and developed with ECL prime (GE Healthcare) according to the manufacturer's instructions. Immunoreactive bands were visualized using LAS-4000mini, and the intensity of bands was quantitated using Science Lab 2006 Multi Gauge software (version 3.X; FUJIFILM, Tokyo, Japan). The following monoclonal or polyclonal antibodies were used: mouse monoclonal anti-GPLD1 antibody (38A1 ab51356-100, Abcam, Cambridge, UK), goat polyclonal anti-GAL3BP antibody (AF2226, R&D Systems, Minneapolis, MN), rabbit polyclonal anti-BACE1 antibody (ab10716, Abcam), rabbit polyclonal anti-ADAM10 antibody (ab1997, Abcam), rabbit polyclonal anti-APP and CTF antibody (A8717, Sigma-Aldrich), mouse monoclonal anti-APP antibody (6E10, Biolegend, San Diego, CA), rabbit polyclonal anti-NRG1 antibody (ab53104 Abcam), rabbit polyclonal antibodies against the carboxyl termini of human sAPPα and sAPPβ (19), mouse monoclonal anti-c-Myc tag antibody (9E10, Santa Cruz Biotechnology, Inc., Dallas, TX), rabbit polyclonal anti-FLAG tag (DDDDK) antibody (PM020, Medical and Biological Laboratories Co., Ltd. (MBL), Nagoya, Japan), and mouse monoclonal anti-β-actin antibody (A1978, Sigma-Aldrich).

### Sandwich ELISAs to detect Aβ40, Aβ42, sAPPα, and sAPPβ

Extracellular Aβ40, Aβ42 sAPPα, sAPPβsw, and βCTF levels in the conditioned media and lysate from H4-APPsw cells were measured using ELISA kits (WAKO Pure Chemical Industries Ltd. and Immuno-Biological Laboratories Co., Ltd., Fujioka, Japan) according to the manufacturer's instructions.

### Cell viability

Extracellular lactate dehydrogenase levels in the conditioned media from H4-APPsw cells were measured using a cytotoxicity detection kit (Roche Applied Science, Mannheim, Germany).

### Identification of GAL3BP as a suppressive factor for Aβ production

The conditioned media from HEK293 cells transfected with or without GPLD1 were concentrated using Amicon Ultra 30-kDa centrifugal filter units (Millipore), and the concentrated and flow-through fractions were collected. The concentrated fractions were incubated with lectin affinity beads (WGA-, WFL-, ConA-, ECA-, and VVA-beads; Vector Laboratory, Burlingame, CA) at 4 °C overnight. Material bound to WFL-beads was eluted with 0.1 m *N*-acetyl-d-galactosamine in TBS. The eluted fractions from the WFL-beads were diluted 5 times with 20 mm phosphate buffer (pH 6.8), subjected to a diethylaminoethyl ion-exchange column, and then eluted with stepwise changed concentrations of NaCl in 20 mm phosphate buffer (pH 6.8). The eluted fraction with 0.5 m NaCl was subjected to SDS-PAGE and silver-stained. The band around 90 kDa was subjected to MS to identify the protein.

### Recombinant GAL3BP

The conditioned media from HEK293 cells that stably expressed GAL3BP with Myc/His tags were subjected to cOmplete^TM^ His tag purification resin (Roche, Basel, Switzerland), and bound materials were eluted with TBS containing 0.5 m imidazole. The solvent of the elution fraction was replaced by PBS and concentrated using Amicon Ultra 30-kDa centrifugal filter units (Millipore). The concentrated fraction was used as a recombinant GAL3BP. The preparations obtained in the same processes from mock-transfected HEK293 cells were used as negative controls (vehicle). We also used a commercially available GAL3BP purified from a mouse myeloma cell line, the NS0-derived human galectin-3BP/MAC-2BP protein Val^19^–Ala^585^ with a C-terminal His_10_ tag (R&D Systems).

### RT-PCR

RNAs from HEK293 cells with or without GPLD1 transfection were prepared with the RN easy-plus-mini kit (Qiagen). The cDNAs were synthesized using SuperScriptIII (Invitrogen). The primers used for β-actin were CACCATTGGCAATGAGCGGTTC and AGGTCTTTGCGGATGTCCACGT. The primers used for GAL3BP were ACAGACCTGCTCCAACTGCT and ACAGGGACAGGTTGAACTGC.

### BACE1 activity assay

β-Secretase activity was determined using a β-secretase fluorometric assay kit (Biovision Inc., Milpitas, CA). BACE1 (extracellular domain of human recombinant C-terminal FLAG-tagged) expressed in HEK293 cells was obtained from Sigma-Aldrich. For inhibition studies, recombinant GAL3BP preparations were pre-incubated with BACE1 in DMEM at 37 °C overnight, and β-secretase activity was measured subsequently.

### Immunoprecipitation of APP

APP proteins were immunoprecipitated from the H4-APPwt or H4-APPsw cell lysates using anti-APP antibody (A8717, Sigma-Aldrich) and Protein A-Sepharose Fast Flow (GE Healthcare). For the detection of the immunoprecipitated APP and exogenously added GAL3BP by Western blotting, anti-APP antibody (6E10, Biolegend) and anti-GAL3BP antibody (AF2226, R&D Systems) were used. For co-immunoprecipitation with GAL3BP, the cDNA encoding c-Myc–tagged GAL3BP was transfected into H4-APPsw cells, and the cell lysates were subjected to immunoprecipitation with anti-Myc tag mAb-agarose (MBL) or anti-APP antibody (A8717, Sigma-Aldrich). As a control, nonimmune mouse IgG1-agarose (MBL) or rabbit nonimmunoglobulin (MBL) was used, respectively.

### In vitro APP processing by BACE1

APP proteins were immunoprecipitated from H4-APPsw cells using anti-APP antibody (6E10) and Protein G–Sepharose 4 Fast Flow (GE Healthcare). The immunoprecipitated materials were pre-incubated with GAL3BP at 37 °C overnight and then incubated with BACE1 (Sigma-Aldrich) and BACE1 inhibitor (KMI 1303, Wako) at 37 °C for 3 h in sodium succinate buffer (pH 4.5). The processing of APP was determined by Western blotting or ELISAs.

### Immunofluorescence analysis

H4-APPsw cells treated with exogenous recombinant GAL3BP-Myc were fixed with 4% paraformaldehyde in PBS, incubated with rabbit polyclonal antibody for the c-Myc tag (A-14, Santa Cruz) and mouse mAb for EEA1 (M176-3, MBL), and then incubated with Alexa 488– and Alexa 546–conjugated secondary antibodies. Cell nuclei were counterstained with 4′,6-diamidino-2-phenylindole (DAPI). Images were acquired using a BZ-9000 microscope (Keyence, Osaka, Japan).

### Proximity ligation assay

PLA Duolink PLA kits (Sigma–Aldrich) were used in accordance with the manufacturer's instructions. Briefly, H4-APPsw cells treated with exogenous recombinant GAL3BP-Myc were fixed with 4% paraformaldehyde in PBS, incubated with rabbit polyclonal antibody for the c-Myc tag (9E10, Santa Cruz Biotechnology) and rabbit polyclonal antibody for APP (A8717, Sigma–Aldrich), and then incubated with PLA reagents containing lyophilized oligonucleotides (plus or minus). After the PLA procedures of ligation, amplification, and incubation with detection solution and substrate solution, cell nuclei were counterstained with DAPI. Images were acquired using a BZ-9000 microscope (Keyence, Osaka, Japan).

### Statistical analysis

The reproducibility of the data presented in the figures and described in the text was confirmed in at least three independent experiments except for the data presented in Fig. 3. The experiments presented in Fig. 3 were repeated twice. All data are shown as mean and S.D. values. Statistical analyses were performed by one-way ANOVA followed by Tukey's post-hoc test. The χ^2^-square test was used for the comparison of cell proportions. *p* < 0.05 was considered statistically significant.

### Data availability

The microarray data have been deposited into ArrayExpress and assigned the accession number E-MTAB-7852.

## Author contributions

T. S., M. K., K. K., H. K., N. Y., K. M., N. I., H. I., and T. T. conceptualization; T. S., M. K., K. K., N. Y., K. M., N. I., and H. I. resources; T. S., M. K., and K. K. data curation; T. S., M. K., and K. K. formal analysis; T. S., M. K., and K. K. investigation; T. S. and M. K. visualization; T. S., M. K., and K. K. methodology; T. S. and M. K. writing-original draft; T. S., M. K., K. K., H. K., N. Y., N. I., H. I., and T. T. writing-review and editing; M. K., H. I., and T. T. funding acquisition.

## Supplementary Material

Supporting Information

## Footnotes

2 The abbreviations used are:

AD: Alzheimer's disease
DAPI: 4′,6-diamidino-2-phenylindole
Aβ: amyloid-β
BACE1: β-secretase 1
APP: amyloid precursor protein
sAPPβ: soluble form of APP
APP-βCTF: APP C-terminal fragment
iPS cell: induced pluripotent stem cell
hiPS cell: human iPS cell
WGA: wheat germ agglutinin
WFL: *Wisteria floribunda* agglutinin
ConA: concanavalin A
ECA: *Erythrina cristagalli* lectin
VVA: *Vicia villosa* lectin
ADAM10: a disintegrin and metalloproteinase domain-containing protein 10
GAL3BP: galectin 3–binding protein
EEA1: early endosome antigen 1
GPI: glycosylphosphatidylinositol
ANOVA: analysis of variance
DMEM: Dulbecco's modified Eagle's medium
DAPI: 4′,6-diamidino-2-phenylindole.
