# Supplementary material for: Galectin 3–binding protein suppresses amyloid-β production by modulating β-cleavage of amyloid precursor protein
Source: J Biol Chem. 2020 Jan 29;295(11):3678–91. doi: 10.1074/jbc.RA119.008703 (PMC7076203; doi:10.1074/jbc.RA119.008703)
Supplement: Supporting Information [file supp_295_11_3678__index.html]

Galectin 3–binding protein suppresses amyloid-β production by modulating β-cleavage of amyloid precursor protein — GAL3BP is a modulator of APP processing — Galectin 3–binding protein suppresses amyloid-β production by modulating β-cleavage of amyloid precursor protein — GAL3BP is a modulator of APP processing — Supporting Information 

# Galectin 3–binding protein suppresses amyloid-β production by modulating β-cleavage of amyloid precursor protein

## Supporting Information

- Supporting Information (to be published online) - Tables and Figures
