# Supplementary material for: Galectin 3–binding protein suppresses amyloid-β production by modulating β-cleavage of amyloid precursor protein
Source: J Biol Chem. 2020 Jan 29;295(11):3678–91. doi: 10.1074/jbc.RA119.008703 (PMC7076203; doi:10.1074/jbc.RA119.008703)
Supplement: Supporting Information [file supp_RA119.008703_144665_3_supp_462446_q4gqpx.pdf]

Supporting Information

Title

Galectin 3–binding protein suppresses amyloid-β production by modulating β-cleavage of amyloid precursor protein

Tsuneyoshi Seki, Motoi Kanagawa, Kazuhiro Kobayashi, Hisatomo Kowa, Naoki Yahata, Kei Maruyama, Nobuhisa Iwata, Haruhisa Inoue, Tatsushi Toda

Contents

Supplementary Table S1: Gene expression analysis.
Supplementary Table S2: Mass spectrometric data for identifying GAL3BP.
Supplementary Fig. S1: Principal component analysis.
Supplementary Fig. S2: Aβ production in H4-APPsw cells after GPLD1 knock-down.
Supplementary Fig. S3: Aβ production in HEK293-APPsw cells.
Supplementary Fig. S4: The GAL3BP mRNA in HEK293 cells.
Supplementary Fig. S5: The expression of GAL3BP in H4-APPsw cells.
Supplementary Fig. S6: Effects of GalNAc on the suppressive activity of GAL3BP for Aβ production in H4-APPsw cells.
Supplementary Fig. S7: Effect of GAL3BP knock-down by single Stealth siRNA on Aβ production.
Supplementary Fig. S8: CBB staining patterns of recombinant GAL3BP preparations.
Supplementary Fig. S9: Effects of GAL3BP treatments on the Aβ production and cell viability.
Supplementary Fig. S10: GAL3BP-induced inhibition of Aβ production in H4-APPwt cells.
Supplementary Fig. S11: GAL3BP-induced inhibition of Aβ production in SH-SY5Y cells.
Supplementary Fig. S12: In vitro BACE1 activity.
Supplementary Fig. S13: Whole gel images of the inhibitory effects of GAL3BP on the processing of immunoprecipitated APP by BACE1.

Supplementary Table S1. Gene expression analysis.

Partek Genomics Suite was used to identify differentially expressed genes before and after the ratio of Aβ42/40 changed. Differentially expressed genes were identified by ANOVA using a false discovery rate < 0.3 and an adjusted $P \leq 0.05$ with an at least 1.3-fold change for multiple-hypothesis testing. A total of 316 genes were detected as candidates correlated with the changes in Aβ production and the Aβ42/40 ratio.

Supplementary Table S2. Mass spectrometric data for identifying GAL3BP.

Nine peptides were identified for the human GAL3GP (LG3BP_HUMAN) by the software MASCOT MS/MS Ions Search. The protonated peptides of ASHEEVEGLVEK (334-345) were detected as both divalent (*m/z* 663.8317) and trivalent (*m/z* 442.8862) ions.

Supplementary Fig. S1. Principal component analysis.

The microarray data using Affymetrix Gene Chip Human Exon 1.0 ST arrays were processed with Partek Genomics Suite. The principal component analysis plot for microarray data shows the different patterns among neuronal cells derived from hiPS cells 201B7 (red), 253G4 (blue), and AD4F-1 (green).

Supplementary Fig. S2. Aβ production in H4-APPsw cells after GPLD1 knock-down.

Two different kinds of Stealth siRNA (HSS 142252 or HSS 142253) were individually transfected into H4-APPsw cells, and the amounts of secreted Aβ40 and Aβ42 were measured in the conditioned media using ELISA assays (n = 3, mean (SD)). P > 0.05 versus control by one-way ANOVA with Tukey's post-hoc test.

Supplementary Fig. S3. Aβ production in HEK293-APPsw cells.

GPLD1 expression vector (+GPLD1) was transfected into HEK293-APPsw cells, and the amount of secreted Aβ40 and Aβ42 were measured in the conditioned media using ELISA assays (n = 3, mean (SD)). Conditioned media from mock-transfected HEK293-APPsw cells were used as controls (Cont.). ***P < 0.001 versus control by one-way ANOVA with Tukey's post-hoc test.

Supplementary Fig. S4. The GAL3BP mRNA in HEK293 cells.

The amount of the GAL3BP mRNA was analyzed by reverse transcription-PCR.

Supplementary Fig. S5. The expression of GAL3BP in H4-APPsw cells.

The cDNA encoding GPLD1 was transfected into H4-APPsw cells. The amount of GAL3BP proteins in the cell culture media and in the cell lysates, and the amount of GPLD1 in the cell lysates, were analyzed by Western Blotting. β-Actin was used as a loading control.

Supplementary Fig. S6. Effects of GalNAc on the suppressive activity of GAL3BP for Aβ production in H4-APPsw cells.

(a) H4-APPsw cells were treated with or without commercially available GAL3BP in the absence or presence of GalNAc (75 mM). The amounts of Aβ40 and Aβ42 in the conditioned media were measured using ELISA assays.
(b) H4-APPsw cells that stably express GAL3BP were treated with GlcNAc (75 mM) or GalNAc (75 mM). The amounts of Aβ40 and Aβ42 in the conditioned media were measured using ELISA assays. PBS was used as a control.
The values in the graphs are presented relative to the corresponding controls (n = 3, mean (SD)). *P < 0.05, **P < 0.01 versus control by one-way ANOVA with Tukey's post-hoc test.

Supplementary Fig. S7. Effect of GAL3BP knock-down by single Stealth siRNA on Aβ production.
Conditioned media were prepared from control HEK293 cells (Cont., lane 1), HEK293 cells with GAL3BP knock-down via single Stealth siRNA (+RNAi, lane 2; HSS 180671 or HSS 180672), and HEK293 cells with GAL3BP knock-down and GPLD1 overexpression (+RNAi, +GPLD1, lane 3), and subsequently added to H4-APPsw cells to test for their suppressive activity on Aβ production. The values in this graph are presented relative to the corresponding controls (n = 3, mean (SD)). *P < 0.05 versus control by one-way ANOVA with Tukey's post-hoc test.

Supplementary Fig. S8. CBB staining patterns of recombinant GAL3BP preparations.
Lane 1, a commercially available GAL3BP; Lane 2, recombinant GAL3BP preparations from HEK293 cells that were transfected with cDNA encoding GAL3BP; and Lane 3, control preparations from mock-transfected HEK293 cells.

Supplementary Fig. S9. Effects of GAL3BP treatments on the Aβ production and cell viability.
H4-APPsw cells were treated with various concentrations of the commercially available GAL3BP, and the amount of secreted Aβ40 and Aβ42 (a) and cell viability (b) were measured by ELISA and LDH assays, respectively.
The values in the graphs are presented relative to the corresponding controls (n = 3, mean (SD)). *P < 0.05, **P < 0.01 versus control by one-way ANOVA with Tukey's post-hoc test.

Supplementary Fig. S10. GAL3BP-induced inhibition of Aβ production in H4-APPwt cells.
The commercially available GAL3BP (5 μg/mL) was added to H4 cells that stably express wild-type APP (H4-APPwt cells), and the amounts of Aβ40 and Aβ42 in the media were measured using ELISA assays.

The values in this graph are presented relative to the corresponding controls (n = 3, mean (SD)). *P < 0.05 versus control by one-way ANOVA with Tukey's post-hoc test.

Supplementary Fig. S11. GAL3BP-induced inhibition of Aβ production in SH-SY5Y cells.
The commercially available GAL3BP (5 μg/mL) was added to SH-SY5Y cell cultures, and the amounts of Aβ40 and Aβ42 in the media were determined.
The values are presented relative to the corresponding controls (n = 3, mean (SD)). ***P < 0.001, *P < 0.05 versus control by one-way ANOVA with Tukey's post-hoc test.

Supplementary Fig. S12. In vitro BACE1 activity.
(a) In vitro BACE1 activity was measured in the presence of recombinant GAL3BP (blue squares). The vehicle (orange squares) and BSA (gray triangles) were used as controls, and the values are presented relative to the vehicle controls. BACE1, 4 units.
(b) In vitro BACE1 activity was measured in the presence of the commercially available GAL3BP (black squares). The controls were vehicle controls (orange squares). The values are presented relative to each control.
(c) In vitro BACE1 activity was measured by changing the amount of BACE1 in the presence the commercially available GAL3BP (black squares). The vehicle (orange squares) and BSA (gray triangles) were used as controls, and the values are presented relative to the vehicle control. The values are presented relative to the vehicle controls. GAL3BP, 10 μg/mL; and BSA, 10 μg/mL.
The values in the graphs are presented relative to the corresponding controls (n = 3, mean (SD)). *P < 0.05, **P < 0.01, ***P < 0.001 versus control by one-way ANOVA with Tukey's post-hoc test.

Supplementary Fig. S13. Whole gel images of the inhibitory effects of GAL3BP on the processing of immunoprecipitated APP by BACE1.
The whole gel images for Figs. 6a and 6c. Upper blots were nitrocellulose membrane to detect βCTFs. Lower blots were PVDF membrane to detect full-length APP.

Gene expression analysis

Gene symbol	p
IQGAP2	1.34E-08
OPN3	1.35E-07
BMS1	2.80E-07
CHGB	5.42E-07
BCMO1	5.47E-07
LRRTM1	8.39E-07
ROR1	9.07E-07
RAB7L1	1.26E-06
GABRG3	1.30E-06
SLC6A11	1.34E-06
CPNE5	1.62E-06
FAM189A2	1.81E-06
TNR	2.21E-06
IL17RD	2.26E-06
ABCC9	2.49E-06
D4S234E	3.28E-06
OPCML	3.32E-06
WBSCR17	3.86E-06
OR1F1	3.89E-06
GRIN2A	3.94E-06
DNAH6	3.96E-06
PPP1R13B	3.99E-06
SYNM	4.36E-06
EIF3D	4.49E-06
ZDHHC14	4.86E-06
CAMK2B	5.71E-06
LAMP3	5.82E-06
MST4	6.18E-06
SEMA6D	7.45E-06
MMD2	7.98E-06
SERPINI1	8.87E-06
SEMA3C	9.69E-06
USP53	9.83E-06
KCND2	9.93E-06
NFIX	1.00E-05
IGSF11	1.05E-05
PTPRT	1.18E-05
MANF	1.51E-05
ENPP1	1.52E-05
NUAK2	1.56E-05
KCND3	1.61E-05
APTX	1.66E-05
CRYM	1.67E-05
SEMA3D	1.72E-05
MLC1	1.73E-05
SLC24A3	1.75E-05
KIAA0319	1.79E-05
LRRC16A	1.80E-05
CHD5	2.14E-05
KDM5A	2.15E-05
MBNL1	2.27E-05
ARHGEF4	2.32E-05
ADAM23	2.32E-05
FAM60A	2.44E-05
MMS19	2.79E-05
LGI1	2.80E-05
TFPI2	2.90E-05
CCDC65	2.94E-05
SLC23A2	2.95E-05
MSRA	3.02E-05
SH3BP5	3.04E-05
SEC23IP	3.09E-05
SEC23A	3.11E-05
ARHGAP29	3.16E-05

Gene symbol	p
CACNG2	3.30E-05
GPLD1	3.53E-05
CYP46A1	3.60E-05
AJAP1	3.62E-05
PCDH10	3.84E-05
IGFBP2	3.85E-05
CHL1	3.90E-05
PITPNC1	4.05E-05
SOX10	4.10E-05
C9orf91	4.11E-05
B3GALT1	4.13E-05
DPP6	4.15E-05
SEMA4B	4.29E-05
AGPAT3	4.33E-05
EPHA6	4.36E-05
GRM4	4.37E-05
KLHL3	4.44E-05
SAMD9L	4.46E-05
SPON1	4.51E-05
NCDN	4.52E-05
MAN2A1	4.58E-05
ADHFE1	4.79E-05
FAM81A	4.80E-05
NANOS1	5.04E-05
PITPNA	5.09E-05
RASSF5	5.10E-05
ST5	5.12E-05
CCDC125	5.22E-05
MFNG	5.30E-05
GNG7	5.31E-05
MYT1L	5.35E-05
PARP11	5.39E-05
KIAA1217	5.45E-05
SLITRK3	5.46E-05
COL12A1	5.58E-05
SMARCD3	5.68E-05
EMB	5.90E-05
C14orf159	5.98E-05
SCN4B	6.45E-05
FAM72D	6.45E-05
CACNB2	6.61E-05
RTTN	6.64E-05
HSP90B1	6.75E-05
SHISA6	6.81E-05
GPR1	6.86E-05
MTFR1	6.94E-05
TMEM2	7.16E-05
WNT7A	7.46E-05
PLS3	7.47E-05
CACNA1D	7.65E-05
CYYR1	7.73E-05
FAM107A	7.74E-05
ATP10B	7.96E-05
CNTN4	7.99E-05
KLF6	8.03E-05
USP44	8.13E-05
C17orf76	8.20E-05
11-Sep	8.44E-05
S1PR1	8.55E-05
SLC20A2	8.58E-05
COL11A1	8.60E-05
SLC16A10	8.62E-05
SYT10	8.67E-05
ANKS1B	8.75E-05

Gene symbol	p
C8orf34	8.93E-05
GABBR2	9.08E-05
SLC22A5	9.18E-05
GRM5	9.29E-05
SLC8A3	9.30E-05
CCDC150	9.41E-05
PFKFB2	9.44E-05
TGIF2	9.63E-05
DIRC2	9.79E-05
DSCAML1	9.93E-05
CPT1A	0.00010128
RGS6	0.00010144
LRP2	0.00010185
MASP1	0.00010205
CXCL14	0.00010266
IGF2BP1	0.00010316
ELP2	0.00010406
CLIC1	0.00010485
HIST1H1C	0.00010559
RPS15A	0.00010794
DNMT3B	0.00010976
SLC19A3	0.0001134
ENO1	0.00011394
TIRAP	0.00011444
MALT1	0.00011498
ALS2CR12	0.00011717
ERC1	0.00011813
SLC4A3	0.00011839
SLC25A24	0.00012139
RGS7	0.00012248
FAM19A5	0.00012282
NMD3	0.00012569
LPPR1	0.00012594
NUP37	0.00012595
MAFB	0.00012606
C1QTNF5	0.00012785
KDELC1	0.0001288
VWA3A	0.00012899
PDE1B	0.00012918
SLC12A5	0.00012943
KCNJ10	0.00013163
DHX33	0.00013354
MITF	0.00013562
SLC35D2	0.00013582
RANBP3L	0.00013714
STMN1	0.00013735
GABBR1	0.00013802
IGF2BP2	0.00013952
CELF4	0.00013986
C21orf88	0.00014192
PTP4A2	0.0001436
AGPAT3	0.00014413
TSHR	0.00014629
MARS	0.00014643
MAMDC2	0.000147
DSCAM	0.00014782
MAL2	0.00014961
3-Mar	0.00014973
FXYD3	0.00015049
LRRC7	0.00015198
CCNH	0.0001521
RIMS2	0.00015321
ARF3	0.00015383
SMAD1	0.00015385

Gene symbol	p
RASSF4	0.00015386
NKAIN2	0.00015427
WASF3	0.00015486
CDCA4	0.00015487
CSRNP2	0.00015519
DEPDC1B	0.00015553
EPB41L4B	0.00015671
PTCHD1	0.00015734
EIF2AK4	0.00015814
CYP4V2	0.00015893
PRDX4	0.00015915
PTTG1	0.00015984
IRF3	0.00016083
KIAA1549	0.0001614
PAK7	0.00016161
LRRC4	0.00016218
ABLIM3	0.00016298
NEUROD6	0.00016357
RECQL	0.000164
ADCY2	0.00016434
DPF3	0.00016639
SLC44A3	0.0001688
SIPA1L1	0.00017006
HIST1H4D	0.00017237
URM1	0.00017398
OLFM2	0.00017827
C10orf28	0.00018085
GCNT4	0.0001812
SYT7	0.00018138
CXCL16	0.00018192
DAG1	0.00018216
GRIN2B	0.00018369
GNL3	0.00018457
PLEKHH2	0.00018509
CCDC135	0.00018534
RAB3IP	0.00018567
CDYL2	0.0001859
ABCG4	0.00018701
MYL6	0.00018839
GOLPH3L	0.00019114
KIF13B	0.00019183
HMGB2	0.0001925
NEK7	0.00019253
BNIP2	0.00019256
TRIM23	0.00019276
MDGA2	0.00019603
EMP2	0.00020952
GRID1	0.00021643
HIST1H2AL	0.00022288
C7orf70	0.00022407
GINS3	0.00022606
PTPRM	0.00022632
GAD2	0.00022782
SLC34A2	0.00023284
TFAP2C	0.00023482
GRIA3	0.00023541
C6orf145	0.00023987
FGF14	0.00024133
MYL9	0.00024195
NRP2	0.00024296
FAM5B	0.00024403
DACT1	0.0002466
NCAPD2	0.00024471
HPCAL4	0.00024684

Gene symbol	p
NUP214	0.00025134
ANXA2	0.00025497
SHC4	0.00025523
MYSM1	0.00025556
MAGEE1	0.00025778
B9D2	0.00026158
GPR155	0.00026989
PDGFC	0.00027118
FLRT2	0.00027215
PCSK5	0.00027404
WNK1	0.00028608
C15orf42	0.00028626
TRAPPC10	0.00028644
ACTC1	0.00029186
QSOX2	0.00029228
NFRKB	0.00029521
ABHD2	0.00029574
PTENP1	0.00029603
CADM1	0.00029804
FAM171B	0.00029886
C3orf32	0.00029939
KHSRP	0.0002999
FBXL17	0.00030619
PRKCZ	0.00030896
PDCD2L	0.00030905
SPAG16	0.0003111
AP2A1	0.00031677
NEGR1	0.00031682
ANKRD45	0.00032114
INO80E	0.00032293
DCLK1	0.00032403
RAP1GAP	0.0003245
IGFBP5	0.00032562
CAMKV	0.00032663
FGF9	0.0003269
LHFPL3	0.00032888
ANO2	0.000329
DLG2	0.00033083
MBNL3	0.00033156
GYS1	0.00033265
NOVA1	0.00033456
PPP3CA	0.00033952
SP1	0.00034449
TBC1D16	0.0003477
SCCPDH	0.00035181
PIK3R3	0.00035505
FAM65B	0.00035222
OLFM1	0.00035261
CLK4	0.00035942
SYT13	0.00036126
PTPN5	0.00036197
PSRC1	0.00037283
LONRF3	0.00037287
HOPX	0.00037293
GNAL	0.00037448
CASQ2	0.00038041
S100A6	0.00039035
SCN3B	0.00039182
UBE3B	0.00039332
AKT1	0.00039624

Supplementary Table S1. Gene expression analysis.

Partek Genomics Suite was used to identify differentially expressed genes before and after the ratio of Aβ42/40 changed. Differentially expressed genes were identified by ANOVA using a false discovery rate < 0.3 and an adjusted P ≤ 0.05 with an at least 1.3-fold change for multiple-hypothesis testing. A total of 316 genes were detected as candidates correlated with the changes in Aβ production and the Aβ42/40 ratio.

Observed *m/z*	Observed Monoisotopic Mass/Da	Calculated Monoisotopic Mass/Da	Difference/ mDa	Error (ppm)	Peptide Sequence	Position	MASCOT Ion Score
796.8965	1591.778	1591.784	-6	4	ELSEALGQIFDSQR	138-151	46
566.3389	1130.663	1130.666	-3	3	RIDITLSSVK	216-225	60
488.2859	974.557	974.565	-8	8	IDITLSSVK	217-225	67
678.3901	1354.766	1354.771	-5	4	SDLAVPSELALLK	311-323	48
442.8862	1325.637	1325.646	-9	7	ASHEEVEGLVEK	334-345	32
663.8317	1325.649	1325.646	3	2	ASHEEVEGLVEK	334-345	44
554.8060	1107.597	1107.604	-7	6	KSQLVYQSR	427-435	43
490.7574	979.500	979.509	-9	9	SQLVYQSR	428-435	26
799.8391	1597.664	1597.669	-5	3	YSSDYFQAPSDYR	442-454	78
841.0610	2520.161	2520.175	-14	6	YYPYQSFQTPQHPSFLFQDK	455-474	22

Table, List of identified peptides for LG3BP_HUMAN

Supplementary Table S2. Mass spectrometric data for identifying GAL3BP.

Nine peptides were identified for the human GAL3GP (LG3BP_HUMAN) by the software MASCOT MS/MS Ions Search. The protonated peptides of ASHEEVEGLVEK (334-345) were detected as both divalent (*m/z* 663.8317) and trivalent (*m/z* 442.8862) ions.

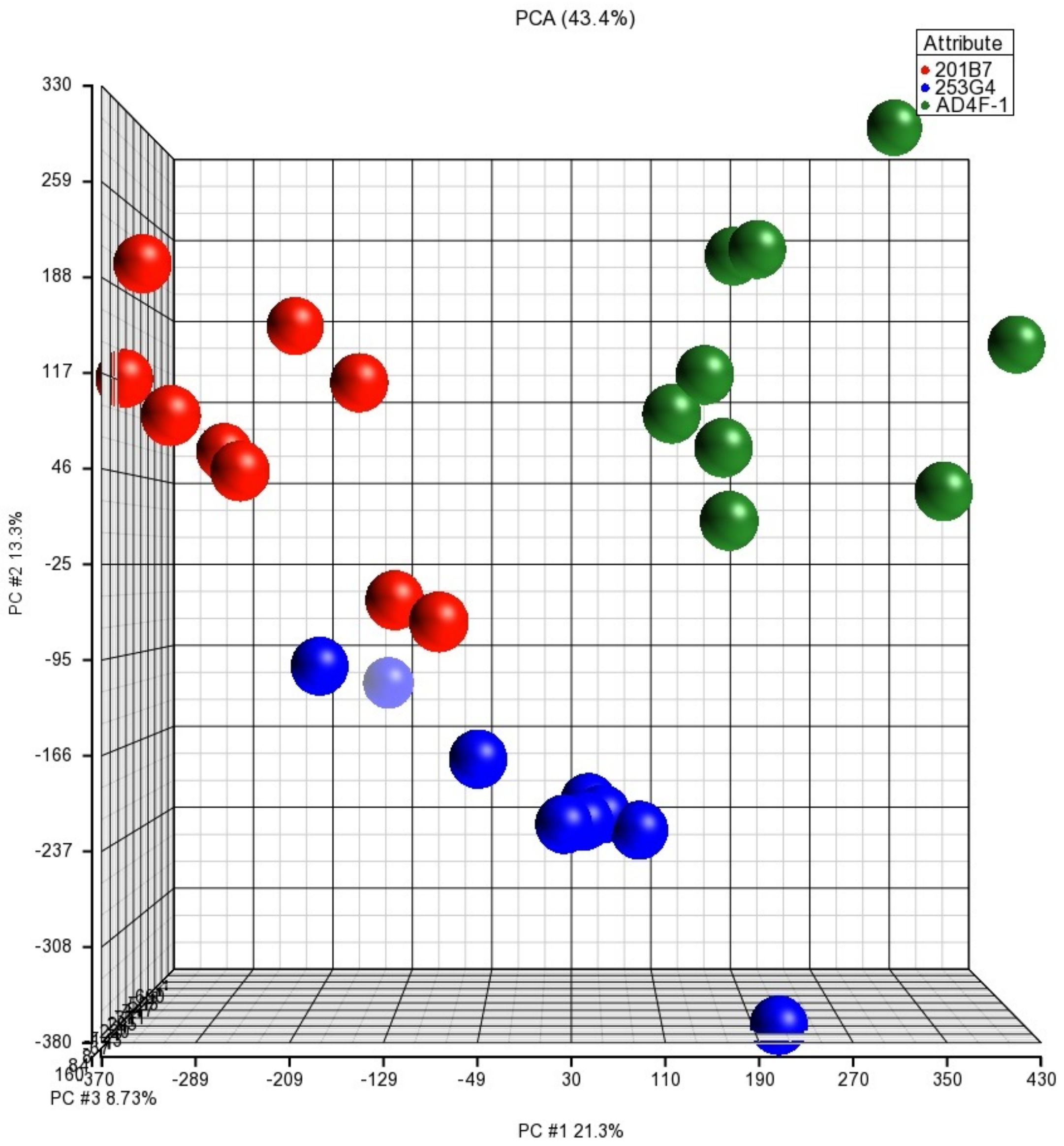

Supplementary Fig. S1. Principal component analysis.

The microarray data using Affymetrix Gene Chip Human Exon 1.0 ST arrays were processed with Partek Genomics Suite. The principal component analysis plot for microarray data shows the different patterns among neuronal cells derived from hiPS cells 201B7 (red), 253G4 (blue), and AD4F-1 (green).

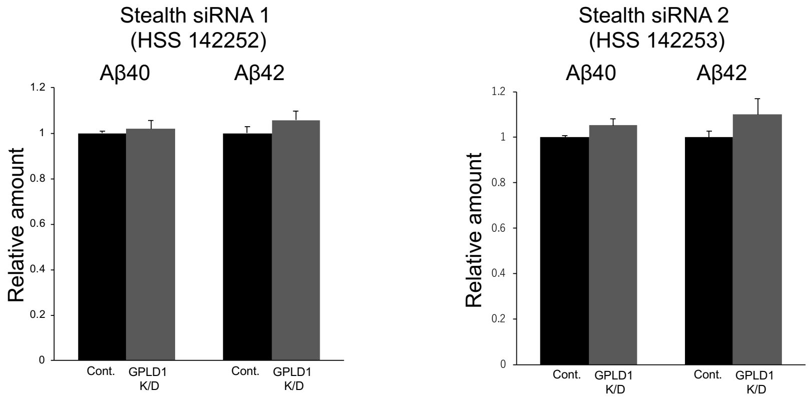

Supplementary Fig. S2. Aβ production in H4-APPsw cells after GPLD1 knock-down. Two different kinds of Stealth siRNA (HSS 142252 or HSS 142253) were individually transfected into H4-APPsw cells, and the amounts of secreted Aβ40 and Aβ42 were measured in the conditioned media using ELISA assays (n = 3, mean (SD)). P > 0.05 versus control by one-way ANOVA with Tukey's post-hoc test.

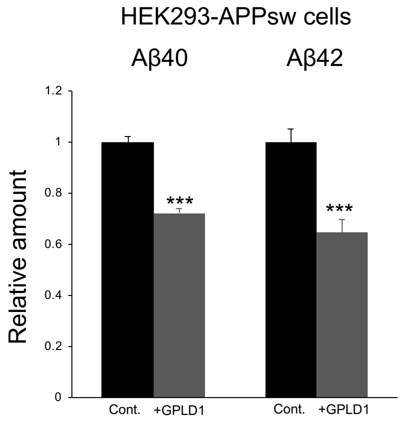

Supplementary Fig. S3. Aβ production in HEK293-APPsw cells.

GPLD1 expression vector (+GPLD1) was transfected into HEK293-APPsw cells, and the amount of secreted Aβ40 and Aβ42 were measured in the conditioned media using ELISA assays (n = 3, mean (SD)). Conditioned media from mock-transfected HEK293-APPsw cells were used as controls (Cont.). ***P < 0.001 versus control by one-way ANOVA with Tukey's post-hoc test.

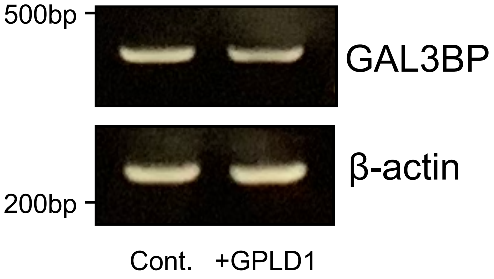

Supplementary Fig. S4. The GAL3BP mRNA in HEK293 cells.

The amount of the GAL3BP mRNA was analyzed by reverse transcription-PCR.

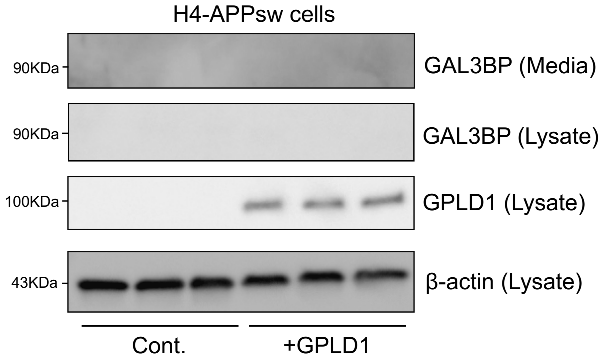

Supplementary Fig. S5. The expression of GAL3BP in H4-APPsw cells.

The cDNA encoding GPLD1 was transfected into H4-APPsw cells. The amount of GAL3BP proteins in the cell culture media and in the cell lysates, and the amount of GPLD1 in the cell lysates, were analyzed by Western Blotting. β -Actin was used as a loading control.

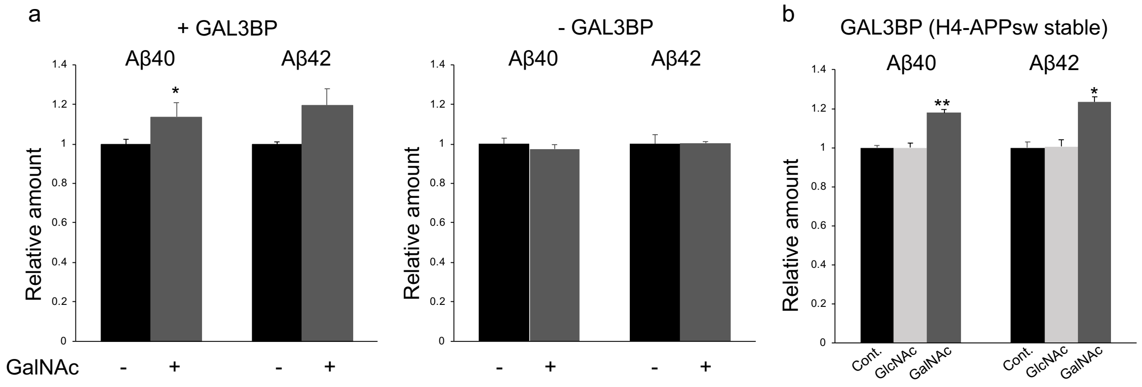

Supplementary Fig. S6. Effects of GalNAc on the suppressive activity of GAL3BP for Aβ production in H4-APPsw cells.

(a) H4-APPsw cells were treated with or without commercially available GAL 3BP in the absence or presence of GalNAc (75 mM). The amounts of Aβ40 and Aβ42 in the conditioned media were measured using ELISA assays.

(b) H4-APPsw cells that stably express GAL3BP were treated with GlcNAc (75 mM) or GalNAc (75 mM). The amounts of Aβ40 and Aβ42 in the conditioned media were measured using ELISA assays. PBS was used as a control.

The values in the graphs are presented relative to the corresponding controls (n = 3, mean (SD)). $^*P < 0.05$, $^{**}P < 0.01$ versus control by one-way ANOVA with Tukey's post-hoc test.

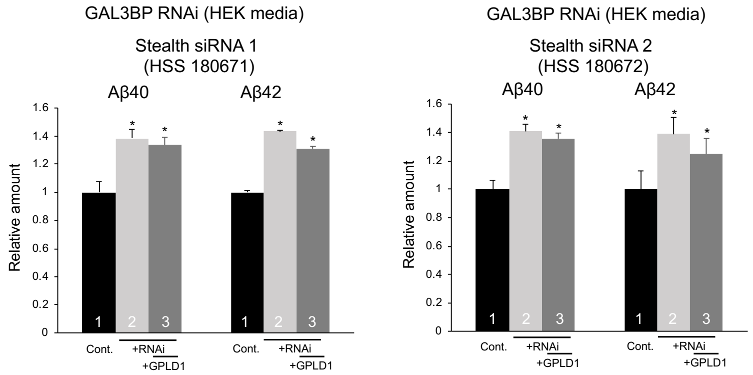

Supplementary Fig. S7. Effect of GAL3BP knock-down by single Stealth siRNA on Aβ production.

Conditioned media were prepared from control HEK293 cells (Cont., lane 1), HEK293 cells with GAL3BP knock-down via single Stealth siRNA (+RNAi, lane 2; HSS 180671 or HSS 180672), and HEK293 cells with GAL3BP knock-down and GPLD1 overexpression (+RNAi, +GPLD1, lane 3), and subsequently added to H4-APPsw cells to test for their suppressive activity on Aβ production. The values in this graph are presented relative to the corresponding controls (n = 3, mean (SD)). *P < 0.05 versus control by one-way ANOVA with Tukey's post-hoc test.

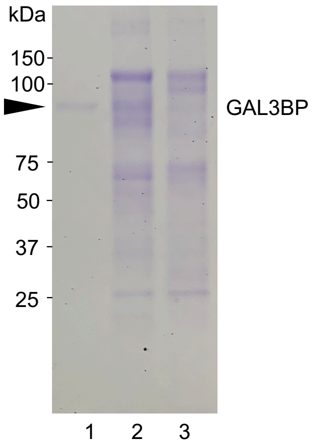

Supplementary Fig. S8. CBB staining patterns of recombinant GAL3BP preparations.

Lane 1, a commercially available GAL3BP; Lane 2, recombinant GAL3BP preparations from HEK293 cells that were transfected with cDNA encoding GAL3BP; and Lane 3, control preparations from mock-transfected HEK293 cells.

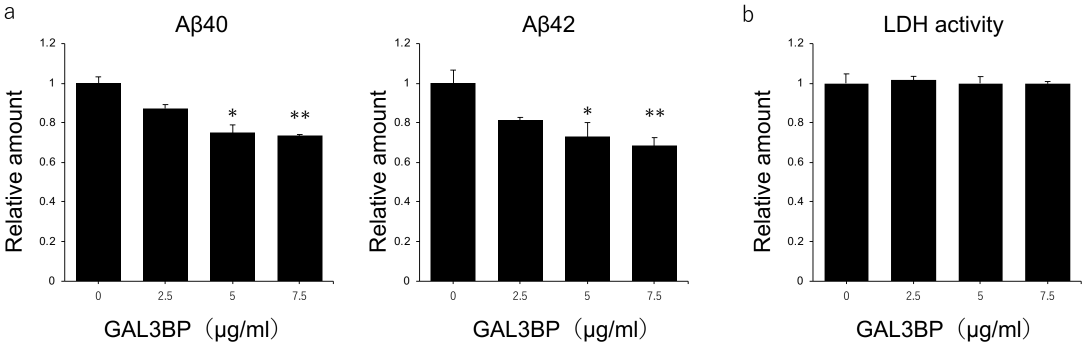

Supplementary Fig. S9. Effects of GAL3BP treatments on the Aβ production and cell viability.

H4-APPsw cells were treated with various concentrations of the commercially available GAL3BP, and the amount of secreted Aβ40 and Aβ42 (a) and cell viability (b) were measured by ELISA and LDH assays, respectively.

The values in the graphs are presented relative to the corresponding controls (n = 3, mean (SD)). *P < 0.05, **P < 0.01 versus control by one-way ANOVA with Tukey's post-hoc test.

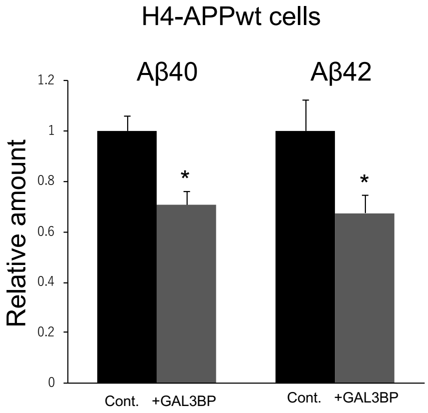

Supplementary Fig. S10. GAL3BP-induced inhibition of Aβ production in H4-APPwt cells. The commercially available GAL3BP (5 μg/mL) was added to H4 cells that stably express wild-type APP (H4-APPwt cells), and the amounts of Aβ40 and Aβ42 in the media were measured using ELISA assays.

The values in this graph are presented relative to the corresponding controls (n = 3, mean (SD)). *P < 0.05 versus control by one-way ANOVA with Tukey's post-hoc test.

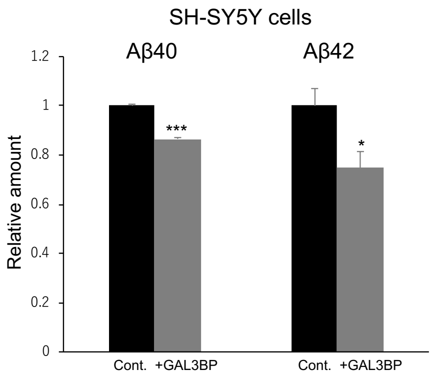

Supplementary Fig. S11. GAL3BP-induced inhibition of A β production in SH-SY5Y cells.
The commercially available GAL3BP (5 μg/mL) was added to SH-SY5Y cell cultures, and the amounts of A β 40 and A β 42 in the media were determined.
The values are presented relative to the corresponding controls (n = 3, mean (SD)). ***$P < 0.001$, *$P < 0.05$ versus control by one-way ANOVA with Tukey's post-hoc test.

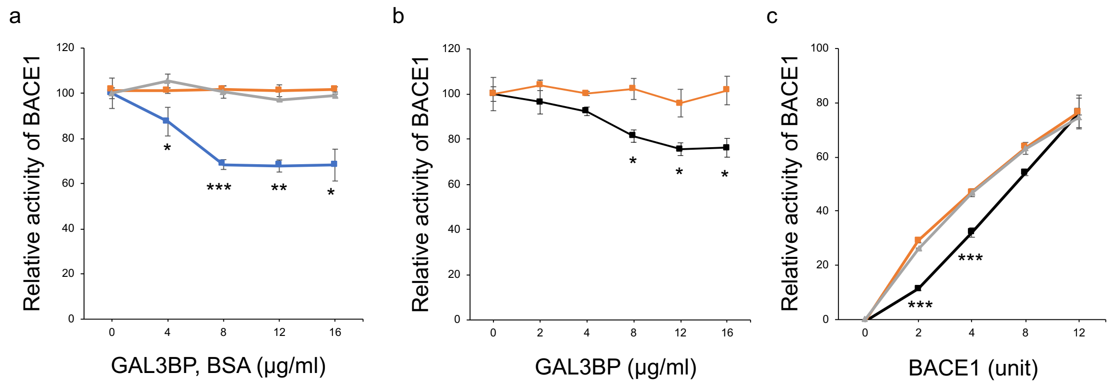

Supplementary Fig. S12. In vitro BACE1 activity.

(a) In vitro BACE1 activity was measured in the presence of recombinant GAL3BP (blue squares). The vehicle (orange squares) and BSA (gray triangles) were used as controls, and the values are presented relative to the vehicle controls. BACE1, 4 units.

(b) In vitro BACE1 activity was measured in the presence of the commercially available GAL3BP (black squares). The controls were vehicle controls (orange squares). The values are presented relative to each control.

(c) In vitro BACE1 activity was measured by changing the amount of BACE1 in the presence the commercially available GAL3BP (black squares). The vehicle (orange squares) and BSA (gray triangles) were used as controls, and the values are presented relative to the vehicle control. The values are presented relative to the vehicle controls. GAL3BP, 10 μg/mL; and BSA, 10 μg/mL.

The values in the graphs are presented relative to the corresponding controls (n = 3, mean (SD)). *P < 0.05, **P < 0.01, ***P < 0.001 versus control by one-way ANOVA with Tukey' s post-hoc test.

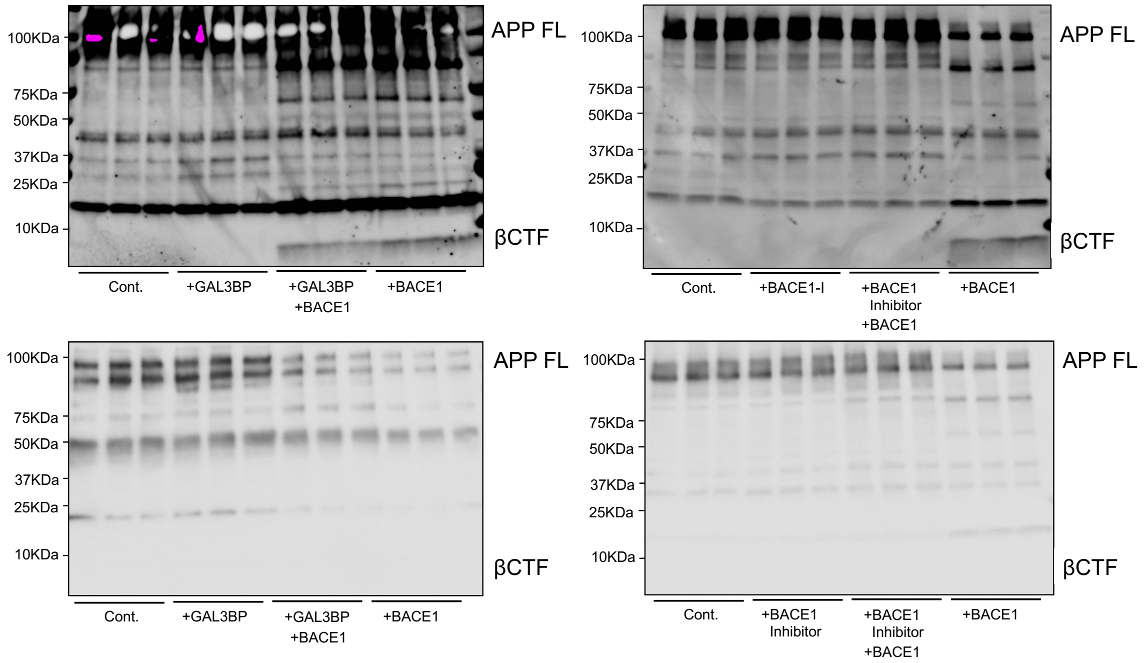

Supplementary Fig. S13. Whole gel images of the inhibitory effects of GAL3BP on the processing of immunoprecipitated APP by BACE1.

The whole gel images for Figs. 6a and 6c. Upper blots were nitrocellulose membrane to detect βCTFs. Lower blots were PVDF membrane to detect full-length APP.
